# The Endogenous GRP78 Interactome in Human Head and Neck Cancers: A Deterministic Role of Cell Surface GRP78 in Cancer Stemness

**DOI:** 10.1038/s41598-017-14604-5

**Published:** 2018-01-11

**Authors:** Hsin-Ying Chen, Joseph Tung-Chieh Chang, Kun-Yi Chien, Yun-Shien Lee, Guo-Rung You, Ann-Joy Cheng

**Affiliations:** 1 Graduate Institute of Biomedical Sciences, Chang Gung University College of Medicine, Tao-Yuan, Taiwan; 2 Department of Radiation Oncology, Chang Gung Memorial Hospital, Tao-Yuan, Taiwan; 3 Proteomics Core Laboratory, Molecular Medicine Research Center, Chang Gung University, Tao-Yuan, Taiwan; 4 Genomic Medicine Research Core Laboratory, Chang Gung Memorial Hospital, Tao-Yuan, Taiwan; 5 Department of Biotechnology, Ming Chuan University, Tao-Yuan, Taiwan; 6 Department of Medical Biotechnology and Laboratory Science, College of Medicine, Chang Gung University, Tao-Yuan, Taiwan

## Abstract

Cell surface glucose regulated protein 78 (GRP78), an endoplasmic reticulum (ER) chaperone, was suggested to be a cancer stem cell marker, but the influence of this molecule on cancer stemness is poorly characterized. In this study, we developed a mass spectrometry platform to detect the endogenous interactome of GRP78 and investigated its role in cancer stemness. The interactome results showed that cell surface GRP78 associates with multiple molecules. The influence of cell population heterogeneity of head and neck cancer cell lines (OECM1, FaDu, and BM2) according to the cell surface expression levels of GRP78 and the GRP78 interactome protein, Progranulin, was investigated. The four sorted cell groups exhibited distinct cell cycle distributions, asymmetric/symmetric cell divisions, and different relative expression levels of stemness markers. Our results demonstrate that cell surface GRP78 promotes cancer stemness, whereas drives cells toward a non-stemlike phenotype when it chaperones Progranulin. We conclude that cell surface GRP78 is a chaperone exerting a deterministic influence on cancer stemness.

## Introduction

Cell surface glucose regulated protein 78 (GRP78) was originally observed during the generation of a vaccine against the avian sarcoma viruses and was thought to be a virus-specific antigen without being named^1^. Shiu *et al*. found that glucose starvation resulted in the up-regulation of this 78-kDa protein in chick embryo fibroblasts, and they concluded that this protein was relevant to glucose metabolism; thus, they named the protein Glucose-Regulated Protein 78^2^. Subsequently, GRP78/BiP was identified as a member of the 70-kDa heat shock protein family^3^ and was found to be involved in immunoglobulin chain synthesis and defective protein degradation in the endoplasmic reticulum^4,5^. Thereafter, several publications showed that GRP78 is a resident chaperone in the endoplasmic reticulum whose physiological function is to facilitate proper protein production. GRP78 overexpression is associated with many physiological and pathological stresses, such as hypoxia, radiation and ultraviolet exposure, immune diseases, low pH condition and tumor malignancies^6–8^.

GRP78 has two reported functions in subcellular compartments: as an endoplasmic reticulum (ER) chaperone in the intracellular compartment, and as a proposed signaling receptor in the plasma membrane (PM) compartment. Several types of malignant cancers express GRP78 on their cell surface^9,10^. Because of this cell surface expression, GRP78 has been hypothesized to function as a signaling receptor that contributes to malignancy. Multiple molecules were previously suggested to associate with, and potentially signal through, cell surface GRP78^11–14^. However, how an ER resident chaperone, which most human cells express, can function as a signaling receptor in a different subcellular compartment is unknown. Additionally, it is unknown whether endogenous GRP78 simultaneously associates with multiple molecules on the cell surface of human cancer cells. Owing to the ER chaperone and stress protein functions of GRP78^6^, many experimental methods, such as gene silencing and exogenous expression, may not be optimal for the elucidation of the physiological and pathological relevance of endogenous GRP78. Therefore, in this study, we examined the endogenous interactome of GRP78 in the subcellular compartments of cancer cells using mass spectrometry and human head and neck cancer (HNC) cell lines as the study model.

GRP78 may play a role in cancer stemness in HNC^15,16^. GRP78 silencing reduces the xenograft tumorigenesis and metastasis in mice and compromises the stem cell niche of mouse hair follicles^15^. Serial dilutions of sorted HNC cells based on cell surface GRP78 levels has a direct influence on xenograft tumorigenesis in mice^16^, suggesting that cell surface GRP78 can influence the self-renewal ability of cancer stem cells. However, the role of cell surface GRP78 in cancer stemness has not been explored in depth. Therefore, we investigated the influence of the association between cell surface GRP78 and the chosen interactome candidate on cell cycle distribution, asymmetric cell division, and expression of stemness associated markers.

## Results

### Detection of the GRP78 Interactome in the Subcellular Compartments of OECM1 and FaDu Cells

**Figure 1:**
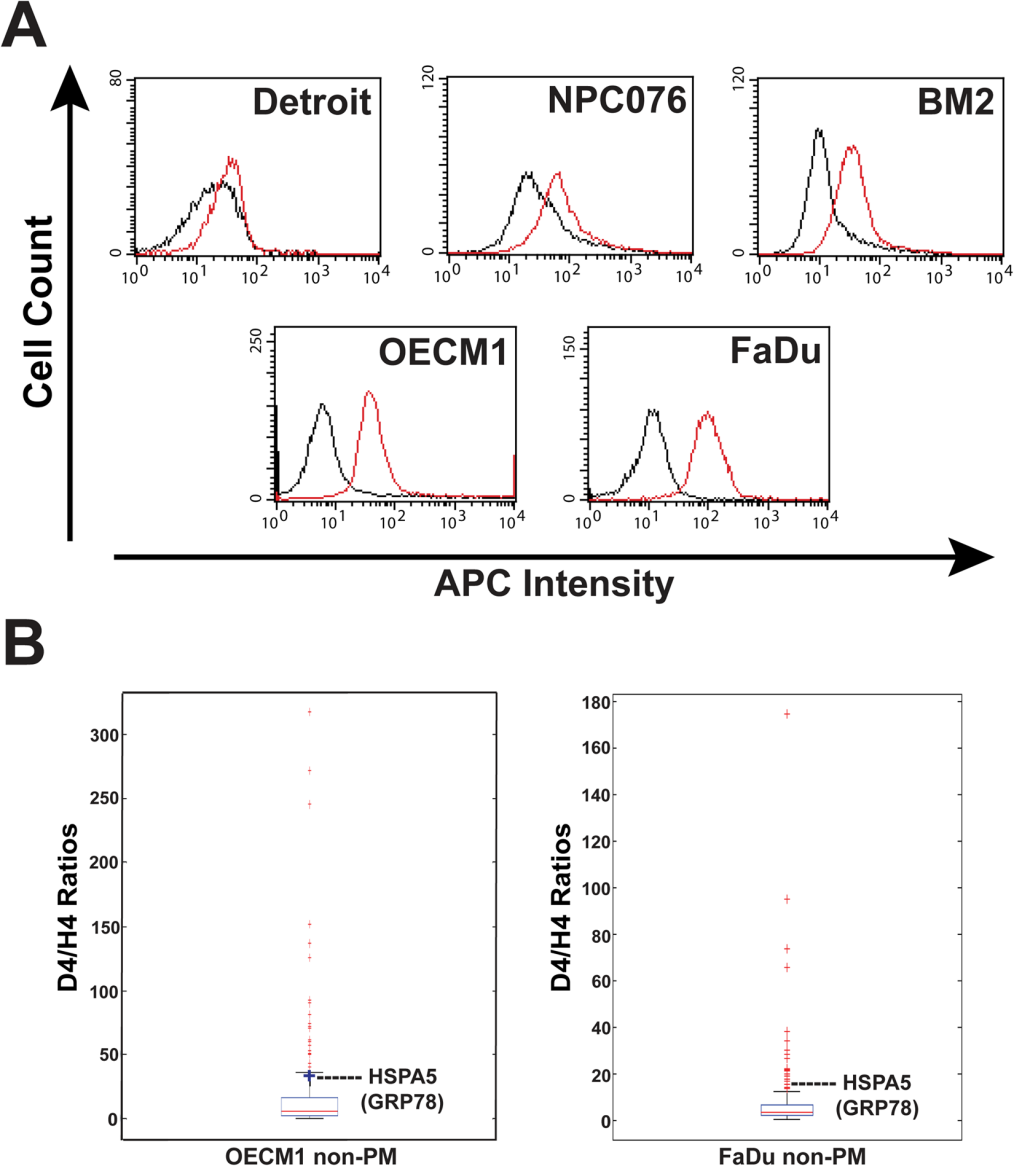
Identification of the endogenous interactome of GRP78 in the non-PM compartment of head and neck cancer cells. (**A**) Different expression levels of GRP78 on the cell surface of 5 head and neck cancer cell lines (Detroit, NPC076, BM2, OECM1, and FaDu) were detected by flow cytometry analysis. The red lines represent signal from the anti-GRP78 antibody, and the black lines represent signal from the isotype control immunoglobulin. (**B**) The endogenous interactome candidates of GRP78 in the non-PM fractions of OECM1 and FaDu with D4/H4 ratios greater than 1.000 were statistically analyzed with box and whisker plots graphed using MATLAB software. In both experiments, GRP78 was successfully detected among the candidates above (for FaDu non-PM) or just below (for OECM1 non-PM) the upper whisker.

GRP78 is not expressed on the cell surface of all tumor cell lines^10^. Therefore, for the detection of the endogenous GRP78 interactome in the PM compartment, the expression levels of cell surface GRP78 in five HNC cell lines were first examined using flow cytometry (Fig. 1A). Because OECM1 and FaDu express higher levels of cell surface GRP78, these cells were chosen for the detection of potential interactome proteins. The cellular proteins were fractionated into 2 subcellular compartments, PM and non-plasma membrane (non-PM, which is the total cell lysate excluding the plasma membrane). After immunoprecipitation using a GRP78 antibody, the co-immunoprecipitated proteins were subjected to two-dimensional liquid chromatography mass spectrometry analysis (2D LC-MS/MS, Supplemental Fig. 1). GRP78 was detected in the non-PM compartments of both OECM1 and FaDu cells (Fig. 1B), demonstrating that our platform is capable of detecting GRP78 and its endogenous interactome. In the non-PM fractions, a total of 191 and 221 interactome proteins (with D4/H4 ratios greater than 1.000) were identified in OECM1 and FaDu cells, respectively. Except keratin-related proteins and GRP78, the top 25 candidates in the non-PM compartments of OECM1 and FaDu cells ranked by their D4/H4 ratios are listed in supplemental Tables S1 and S2. A total of 10 and 18 interactome candidates were identified in the PM fractions of OECM1 and FaDu cells, respectively (Supplemental Table S3). Seven of the PM interactome candidates from FaDu cells and four from OECM1 cells were chosen for subsequent verification experiments, because the known functions of these molecules are potentially relevant to cancer malignancy.

### Verification of Chosen Interactome Candidates of GRP78 in the Plasma Membrane Compartment of OECM1 and FaDu Cells

**Figure 2:**
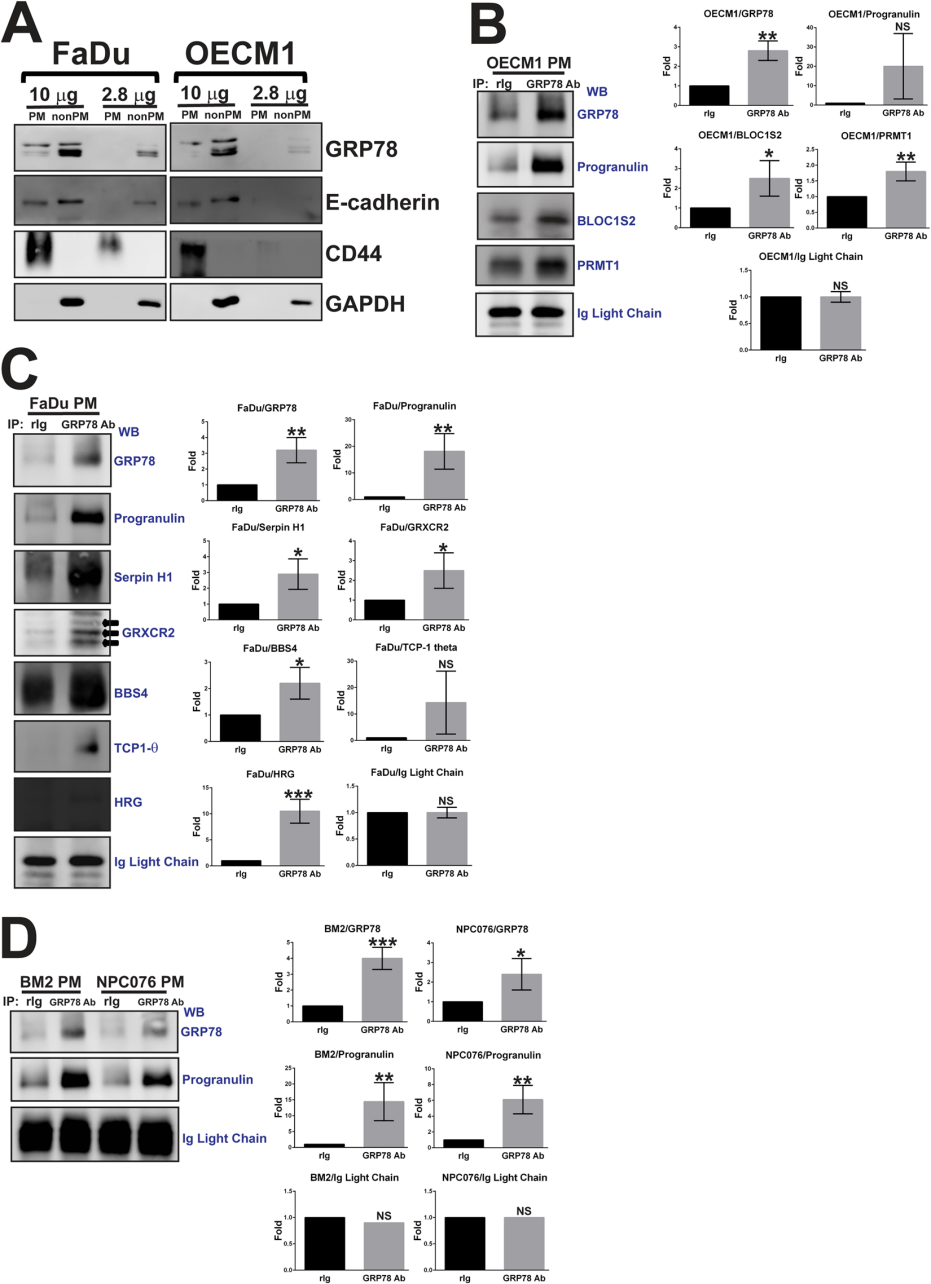
Verification of the endogenous interactome of GRP78 in the PM compartment of OECM1 and FaDu cells by immunoprecipitation and western blot analysis. (**A**) PM and non-PM fractionations were examined by western blot analysis using two membrane markers and one cytosolic marker. The input control for co-immunoprecipitation of GRP78 was also examined with 2 amounts of protein loading. A total of nine GRP78 interactome candidates from the PM compartment were chosen for verification with co-immunoprecipitation and western blot analysis. (**B**) Three interactome candidates co-immunoprecipitated with cell surface GRP78 in OECM cells, and (**C**) six in FaDu cells. (**D**) Progranulin also co-immunoprecipitated with cell surface GRP78 in BM2 and NPC076 cells. Protein bands of all of the blots were quantified using ImageJ software. The expression level of GRXCR2 in FaDu cells (**C**) was determined by quantifying potential isoform bands pointed by three solid arrows. The bar graphs in (**B**) and (**C**) were the mean ± SEM quantified from 3 Western Blots of 3 independent co-immunoprecipitation experiments, and that in (**D**) 3 Western Blots of 2 independent experiments. Full-length blots are presented in Supplementary Figures S5–S8. *p < 0.1, **p < 0.05, and ***p < 0.01; NS = not significant.

The relative amounts of GRP78 in the proteome of the PM and non-PM fractions are consistent with our expectation that GRP78 is more abundant in the non-PM compartment than in the PM compartment (Fig. 2A). Exclusive detection of cell surface and cytosolic markers (CD44 and GAPDH, respectively) demonstrate complete fractionation of the PM and non-PM compartments (Fig. 2A). To verify the association between cell surface GRP78 and the interactome candidates, the PM proteins of OECM1 and FaDu cells that co-immunoprecipitated with GRP78 were subjected to western blot analysis. Progranulin is the precursor of Granulins^17^ (accession number P28799; Supplemental Table S3). Because the peptide fragments ionized by the mass spectrometer are generated from protein digestion, it is reasonable that after protein fragmentation, Progranulin is detected as Granulins by the Mascot database-matching algorithm. In the PM compartments, three proteins (Progranulin, BLOC1S2, and PRMT1) were confirmed to be associated with GRP78 in OECM1 cells (Fig. 2B), and six (Progranulin, Serpin H1, GRXCR2, BBS4, TCP-1 theta, and HRG) were confirmed to be associated with GRP78 in FaDu cells (Fig. 2C). Complement 4-alpha did not co-immunoprecipitate with cell surface GRP78 in the OECM1 and FaDu cell PMs (data not shown). Although BM2 and NPC076 cells have lower expression levels of cell surface GRP78 (Fig. 1A), Progranulin also associated with cell surface GRP78 in these two cell lines (Fig. 2D).

**Figure 3:**
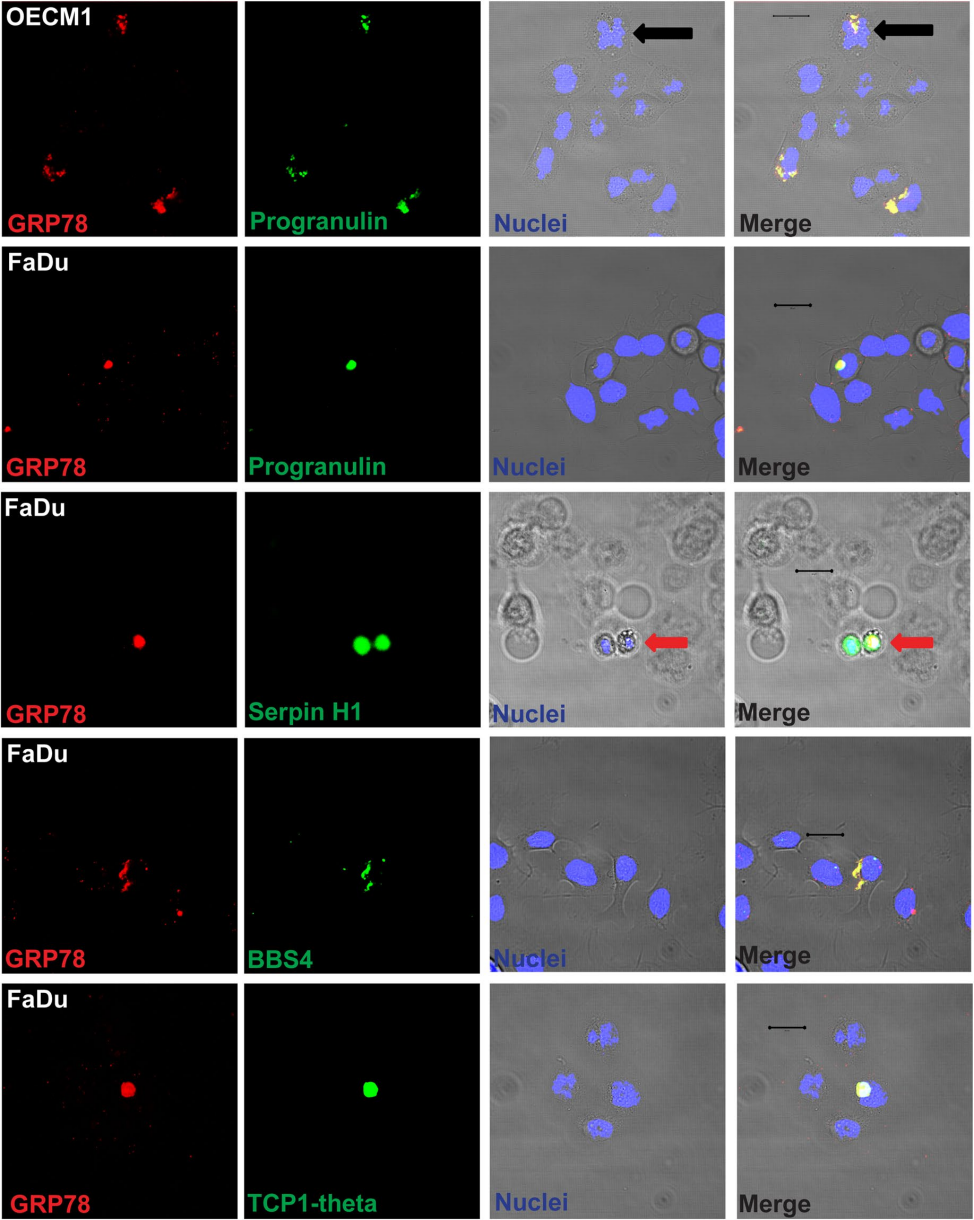
Verification of the GRP78 interactome candidates in the PM compartment of OECM-1 and FaDu cells by confocal microscopy. The interactome candidates that co-immunoprecipitated with cell surface GRP78 were further confirmed by immunostaining OECM1 and FaDu cells, followed by confocal microscopy. The pseudo-color red in all images reflects the immunofluorescence signal from GRP78, and the pseudo-color green reflects the immunofluorescence signal from the interactome candidates. The nuclei were stained with Hoechst 33342 and appear as blue signal. The black and red arrows point to cells in different stages of division. Progranulin and BBS4 are interactome candidates on the cell surface, and Serpin H1 and TCP-1 theta are interactome candidates at the intracellular side of the PM. The fluorescence images were acquired using a 63X oil lens on an LSM780 confocal microscope. Scale bars = 20 μm.

To confirm these interactome candidates of cell surface GRP78, confocal microscopy was used to examine the co-localization between interactome candidates and cell surface GRP78. The immunofluorescence staining of Progranulin, Serpin H1, BBS4, and TCP-1 theta perfectly overlapped with that of GRP78 (Fig. 3), suggesting that GRP78 and these interactome candidates co-localize in the PM compartment. Only partial overlap was observed between the fluorescence staining of GRXCR2/GRP78, HRG/GRP78, BLOC1S2/GRP78, and PRMT1/GRP78 (Supplemental Fig. S2). Complement 4 alpha did not co-localize with GRP78 (data not shown). Notably, the co-localization between Progranulin and GRP78 occurred at the cleavage furrow of a dividing OECM1 cell during telophase (black arrows, Fig. 3). Moreover, GRP78 was asymmetrically expressed by one of the daughter cells that had just completed the process of cell division in the FaDu cell line (red arrows, Fig. 3).

Progranulin is a spermatocyte-specific glycoprotein expressed meiotically and post-meiotically in the acrosomes of guinea pigs^18^. Progranulin is also transiently expressed in oocytes, at the morula stages and in the trophectoderm of mouse preimplantation embryos^19^ and is involved in the differentiation of rat brain cells^20^ and human osteoclasts^21^. Pathologically, Progranulin promotes *in vitro* and *in vivo* tumorigenesis of poorly tumorigenic epithelial cancer cells^22,23^ and contributes to the regulation of cell cycle progression and cell division^24–26^. Additionally, Progranulin was detected in the PM compartment of both OECM1 and FaDu cells. Therefore, the influence of cell population heterogeneity on cancer stemness according to the cell surface levels of GRP78 and Progranulin was further investigated.

### Cells with Different Surface Levels of GRP78 and Progranulin Exhibit Distinctive Cell Cycle Distributions

**Figure 4:**
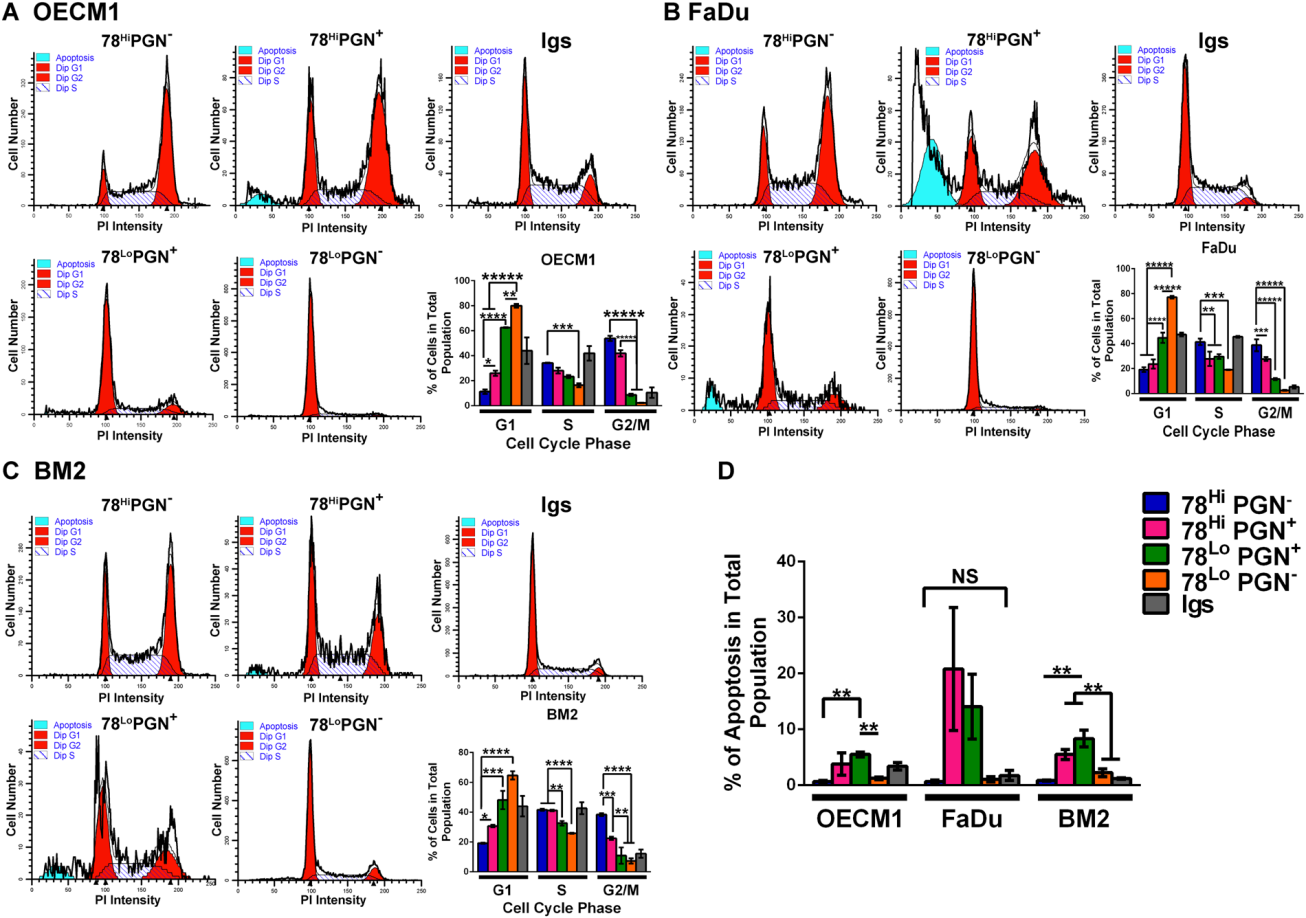
The cell cycle distributions of four cell groups sorted according to cell surface levels of GRP78 and Progranulin. Four sorted cell groups of OECM1 (**A**), FaDu (**B**), and BM2 (**C**) show very similar trends of cell cycle distributions. One representative set of cell cycle histograms of OECM1, FaDu, and BM2 cells is shown. The Igs group was cells incubated with the isotype control immunoglobulins corresponding to the antibodies recognizing GRP78 and Progranulin; this group represents the sham sorted total population. Data from the sorted OECM1 groups in the bar graph are calculated as the mean ± SEM of three replicates from two independent sorting experiments, and that from the sorted FaDu groups three replicates three independent experiments, and sorted BM2 groups three replicates two independent experiments. (**D**) Although the rates of apoptosis among the five groups of FaDu cells are not significantly different by multiple comparison tests, a p-value < 0.1 was obtained from one-way ANOVA. *p < 0.1, **p < 0.05, ***p < 0.01, ****p < 0.001, and *****p < 0.0001; NS = not significant.

The co-localization between Progranulin and GRP78 at the cleavage furrow of a dividing OECM1 cell during telophase (black arrows, Fig. 3) encouraged us to examine the influence of cell population heterogeneity on cell cycle distribution in HNC cells according to cell surface levels of GRP78 and Progranulin (PGN). The total populations of OECM1, FaDu, and BM2 cells were sorted into four cell groups (GRP78^Hi^PGN^−^, GRP78^Hi^PGN^+^, GRP78^L^°PGN^+^, and GRP78^L^°PGN^−^) according to the cell surface levels of GRP78 and Progranulin (Supplemental Fig. S3). Among the sorted four groups of these three cell lines, the percentages of GRP78^Hi^ cells in the G2/M phase were significantly higher than that of GRP78^L^° cells. Consistently, the percentages of GRP78^L^° cells in the G1 phase were significantly higher than that of GRP78^Hi^ cells (Fig. 4A,B, and C, bar graphs). Additionally, the percentages of GRP78^Lo^PGN^+^ cells in the G1 phase were significantly lower than that of GRP78^Lo^PGN^−^ cells (Fig. 4A,B, and C, bar graphs), demonstrating that both cell surface GRP78 and Progranulin, whether associated to each other or expressed alone, can drive OECM1, FaDu, and BM2 cells beyond the G1 phase. Although the GRP78^Hi^PGN^+^ groups of OECM1, FaDu, and BM2 cells also showed higher percentages of cells in the G2/M phase than the GRP78^Lo^PGN^+^ groups (Fig. 4A,B, and C, bar graphs), various levels of apoptosis were observed in the PGN^+^ cells of these three cell lines (Fig. 4D). Pluripotency of human embryonic stem cells is maintained in the S and G2 phases and is regulated independently of the G1 phase^27^. Our cell cycle distribution results demonstrate that cell surface GRP78 expression levels significantly correlates with the maintenance of cancer stemness, and Progranulin may have a role in this process. These results indicate that the association between GRP78 and Progranulin plays an important role in cell fate determination, and may be a critical event for determining whether cancer stem cells commence the reprogramming process.

### Differential Patterns of Symmetric and Asymmetric DNA Distribution in HNC Cells of Different Cell Surface Levels of GRP78 and Progranulin

The asymmetric expression of cell surface GRP78 observed in one of the daughter cells during late telophase (Fig. 3, red arrows) prompted us to examine whether cell surface GRP78 exerts an influence on asymmetric cell division, a hypothetically crucial process for maintaining cancer stemness^28–30^. Previously, the correlation between high/low asymmetric DNA segregation and the percentage of CD44^+^CD24^−/lo^ cells in basal-like and luminal human breast cancer cells was investigated^30^. For a qualitative and quantitative assay of higher throughput, we modified the original experimental approach^29^ by using flow cytometry to examine the relationship between asymmetric/symmetric DNA distribution and the heterogeneity of the cell population based on the cell surface levels of GRP78 and Progranulin in OECM1, FaDu, and BM2 cells.

**Figure 5:**
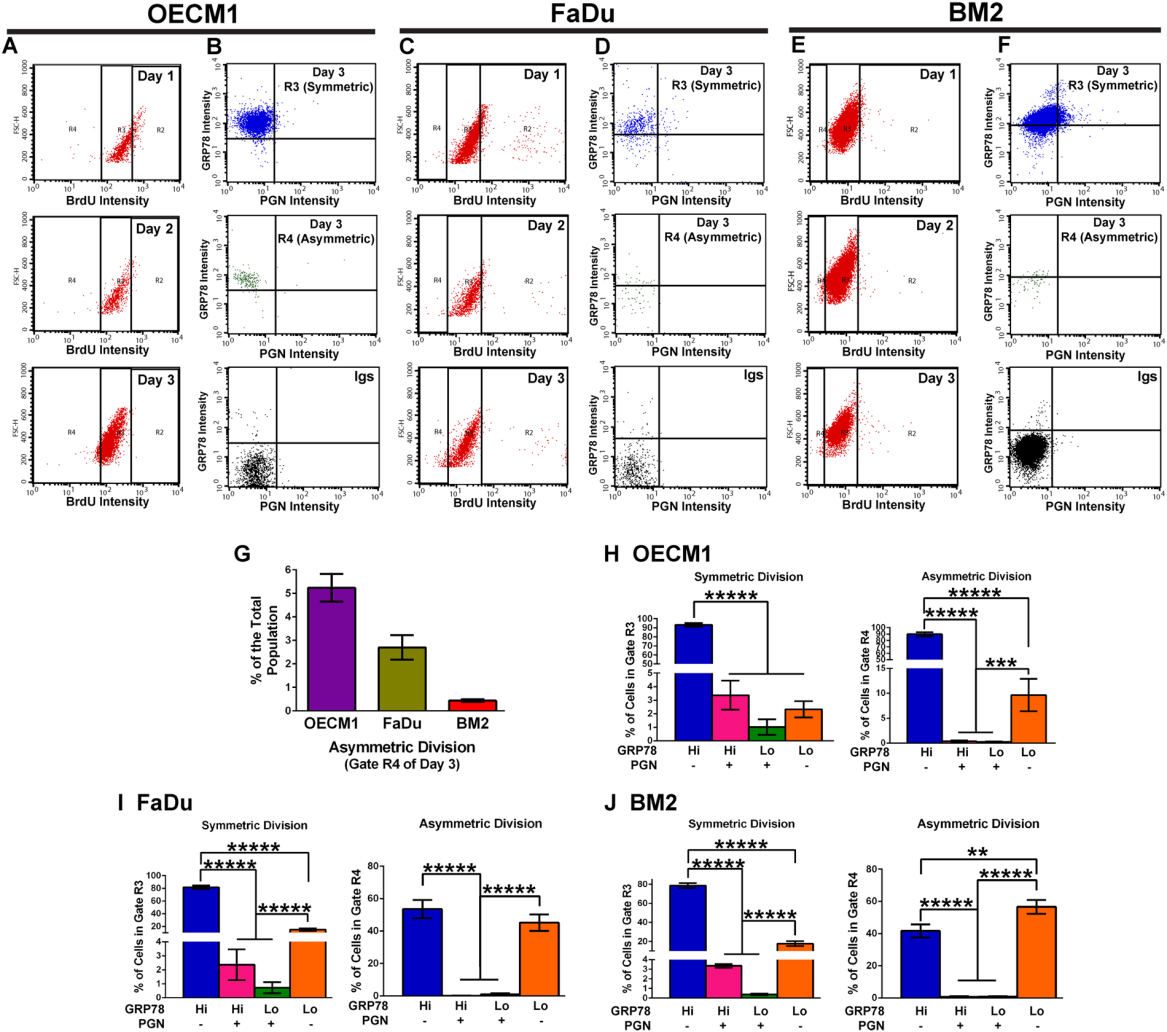
Differential patterns of symmetric and asymmetric DNA distribution in cells with different surface levels of GRP78 and Progranulin. Day 1 was the last day of BrdU labeling, and days 2 and 3 were after BrdU withdrawal for 24 and 48 hours, respectively. (**A**,**C**, and **E**) The levels of BrdU labeling in OECM1, FaDu, and BM2 cells during the three days were compared to distinguish symmetric (gate R3) and asymmetric (gate R4) cell division. (**B**,**D**, and **F**) The cell surface expression levels of GRP78 and Progranulin in R3 and R4. (**G**) The percentages of asymmetric cell division in the total populations of OECM1 and FaDu cells were within the range of 1–5%, and that of BM2 cells below 1%. (**H**,**I**, and **J**) Based on the cell surface levels of GRP78 and Progranulin (PGN), the population heterogeneity of the symmetric (R3) and asymmetric (R4) cell division are represented as bar graphs. Data were calculated as the mean ± SEM of 10 and 8 replicates from 3 independent experiments with OECM1 and FaDu cells, respectively. Eight replicates from 2 independent experiments with BM2 cells were also calculated as mean ± SEM. **p < 0.05, ***p < 0.01, ****p < 0.001, and *****p < 0.0001.

OECM1, FaDu, and BM2 cells were harvested on the last day of BrdU labeling (Day 1), as well as 24 (Day 2) and 48 (Day 3) hours after BrdU withdrawal. As expected, the BrdU-FITC intensity gradually decreased from Day 1 to Day 3 (Fig. 5A,C, and E). The dot plots of forward/- side scatter versus BrdU-FITC intensity of the Day 2 cells were used as the gating standard for determining the highest (gate R2), medium (gate R3) and lowest levels (gate R4) of BrdU incorporation, because the majority of Day 2 cells had undergone one cell division and displayed a medium level of BrdU incorporation (i.e., the most homogenous FITC level among those three days; Fig. 5A,C and E). On Day 3, the cells within gate R3 maintained most of the BrdU incorporation and thus represented cells that underwent symmetric cell division. The cells within gate R4 on Day 3 lost most BrdU labeling, which represents that these cells underwent asymmetric (or lowly symmetric) cell division (Fig. 5A,C, and E). After BrdU withdrawal for 2 days, 5.24 ± 0.59%, 2.70 ± 0.52%, and 0.44 ± 0.05% of the total cell populations of OECM1, FaDu, and BM2, respectively, were in gate R4 (Fig. 5G), showing that asymmetric cell divisions occurred in our cell populations.

The percentages of the four cell subpopulations determined by cell surface levels of GRP78 and Progranulin in gates R3 and R4 on Day 3 (Fig. 5B,D and F) were calculated (Fig. 5H,I, and J). Most of the OECM1 cells in gates R3 and R4 were GRP78^Hi^PGN^−^ (Fig. 5H), and this trend was partially shown in the FaDu and BM2 cells (Fig. 5I and J). All of the Progranulin-expressing OECM1 cells (including cells of GRP78^Hi^PGN^+^ and GRP78^Lo^PGN^+^; 4.39 ± 1.64% in total) were in gate R3, not R4, demonstrating that Progranulin-expressing cells did not undergo asymmetric cell division but only underwent symmetric cell division (Fig. 5H, left). FaDu and BM2 cells expressing Progranulin also displayed a very similar trend (Fig. 5I and J, left). This assay demonstrates that HNC cells expressing cell surface GRP78 exhibit higher percentages of both symmetric and asymmetric cell division, whereas cells expressing Progranulin exclusively undergo symmetric cell division.

### Cell Surface Expression of GRP78 Directs Cells toward Cancer Stemness and Drives Cells Away from Stemness When It Chaperones Progranulin

During embryogenesis, tumorigenesis, and carcinogenesis, cells are often in a niche experiencing various types of stress. GRP78 is a common chaperone expressed in most human cells in response to various physiological and pathological stresses^6,31–33^. Therefore, cell surface expression of GRP78 may represent a crucial cellular stress response that maintains cancer stemness. To test this hypothesis, we quantified the relative normalized expression of markers associated with stemness (Nanog, Oct4, Sox2, TERF-1, and PRDM14) of the four sorted groups of OECM1, FaDu, and BM2 cells by quantitative RT-PCR.

**Figure 6:**
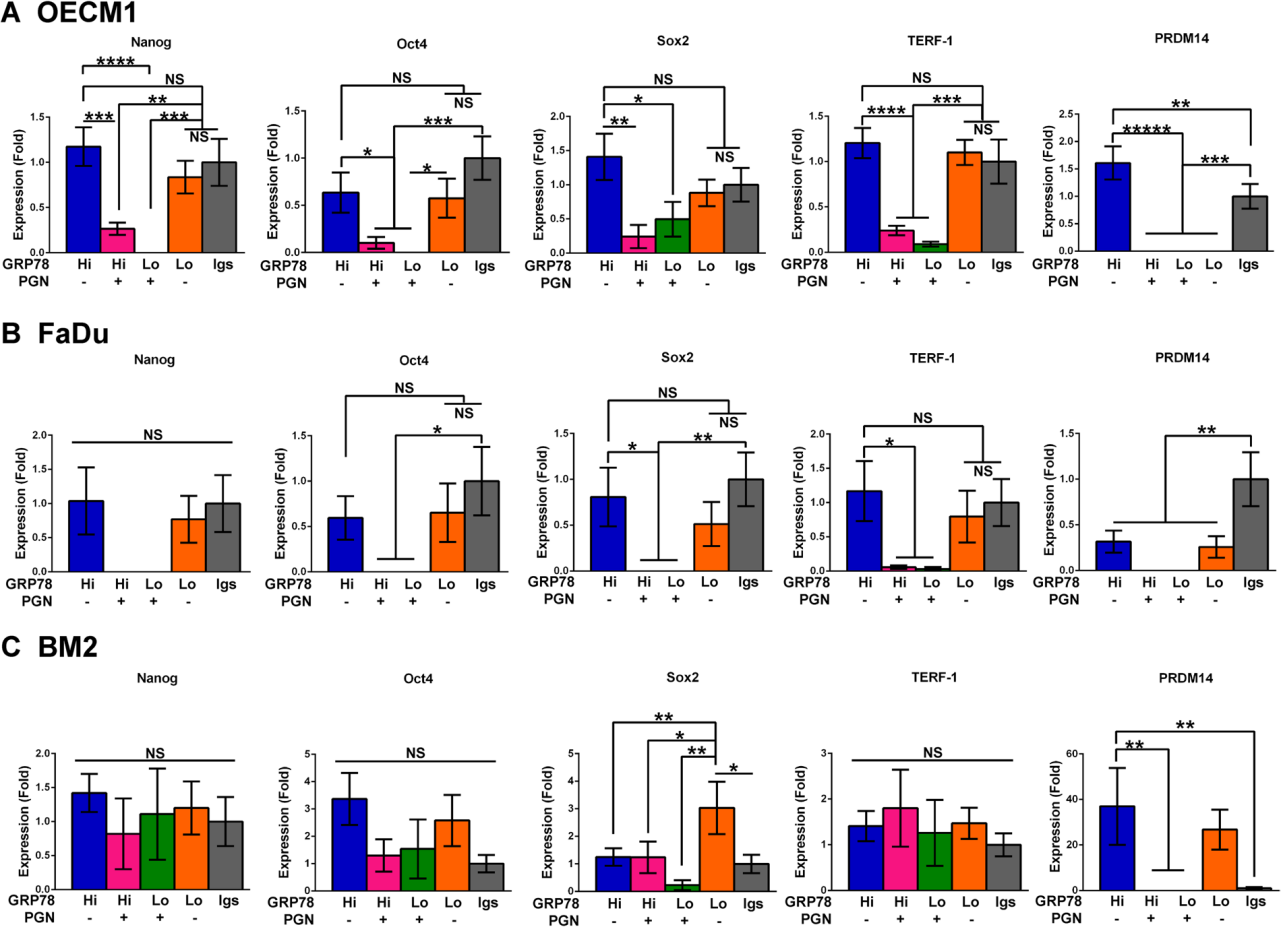
The expression levels of stemness associated markers in OECM1, FaDu, and BM2 cells with different cell surface levels of GRP78 and Progranulin. The relative expression levels of five stemness-associated markers (Nanog, Oct4, Sox2, TERF-1, and PRDM14) in the five sorted groups of OECM1 (**A**), FaDu (**B**), and BM2 (**C**) cells were determined by RT-qPCR. The relative normalized expression levels of these five markers are consistently down-regulated in the GRP78^Hi^PGN^+^ and GRP78^Lo^PGN^+^ groups of OECM1 and FaDu cells. Data in each bar graph were presented as the mean ± SEM of five replicates from the two independent sorting experiments of OECM1, FaDu, and BM2 cells; *p < 0.1, **p < 0.05, ***p < 0.01, ****p < 0.001, and *****p < 0.0001; NS = not significant.

In OECM1 cells, the relative expression of these 5 markers in the GRP78^Hi^PGN^−^ group were significantly higher than or comparable to the Igs group (which consisted of cells incubated with the isotype control immunoglobulins corresponding to the antibodies recognizing GRP78 and Progranulin; this group represents the sham sorted total population), demonstrating that this subpopulation is responsible for the expression of the stemness-associated markers (Fig. 6A). Except for PRDM14, the levels of the other 4 stemness-associated markers in GRP78^Lo^PGN^−^ cells were not significantly lower than the GRP78^Hi^PGN^−^ and Igs groups (Fig. 6A), suggesting that OECM1 cells expressing lower levels of cell surface GRP78 already express these stemness-associated markers, or that the levels of these markers can be sustained for a certain period of time after the levels of cell surface GRP78 have decreased. This trend was also consistently observed in the FaDu cells, with the exception of PRDM14 (Fig. 6B). Among the four groups of sorted OECM1 cells, PRDM14 is exclusively expressed by GRP78^Hi^PGN^−^ cells, indicating a strong influence of cell surface GRP78 on the up-regulation of PRDM14 for cancer stemness (Fig. 6A). Regardless of the levels of cell surface GRP78, all five markers were consistently down-regulated or not detectable in the Progranulin-expressing OECM1 and FaDu cells, demonstrating that cancer stemness decreases when cell surface GRP78 chaperones Progranulin (Fig. 6A and B). As to the four groups of sorted BM2 cells, only the expression levels of Sox2 and PRDM14 shows statistically significant results, although that of Nanog and Oct4 also have partial similar trend to sorted OECM1 and FaDu cell groups (Fig. 6C). It is very interesting that the GRP78^Lo^PGN^−^ group of BM2 cells had significantly highest level of Sox2 among the 5 sorted groups (Fig. 6C). Perhaps it indicates that the turnover rate of Sox2 in HNC cells with lower levels of cell surface GRP78 is fast.

After GRP78 knockdown, Progranulin no longer expressed in the PM compartment of OECM1 cells (Fig. S4A). Although it did not affect cell cycle distribution of OECM1 cells (Fig. S4B), GRP78 silencing significantly reduced the expression levels of all of the five stemness associated markers (Fig. S4C). GRP78 knockdown greatly compromised the proliferation capability of OECM1 cells, so asymmetric cell division assay cannot be performed in GRP78 silenced cells.

## Discussion

Since cell surface GRP78 was hypothesized to function as a signaling receptor and a cancer stem cell marker, many research groups have proposed its use as a therapeutic target. According to our results describing the GRP78 interactome in the PM compartment, cell surface GRP78 associates with multiple molecules in both OECM1 and FaDu cells; however, most signaling receptors do not simultaneously bind to many ligands. Therefore, our finding demonstrates that cell surface GRP78 serves as a resident chaperone for the synthesis of a part, if not all, of the proteome in the PM compartments of OECM1 and FaDu cells, rather than functioning as a signaling receptor. The expression levels of cell surface GRP78 varies among different tumor cell types and tumor cell lines. Most tumor cell lines are established from the clinical specimens of tumor patients, including the 5 HNC cell lines used in this article. GRP78 was previously reported to be relevant to malignant phenotypes of head and neck cancers, and we described a deterministic role of cell surface GRP78 as a chaperone for cancer stemness in this article. Therefore, it is likely that different levels of cell surface GRP78 among tumor cell lines represent various stages of tumor development or progression. It might also directly or indirectly reflect the specific reprogramming phase, the achieved extent of cancer stemness, or the dysregulated order of cell fate determination, of a tumor cell type/line.

One of the characteristics of cancer stem cells is their low percentage (1~5%) in a cell population. However, because more than 50% of the cells express cell surface GRP78 in OECM1, FaDu, and BM2 cell lines, our data conflict with the characteristics of typical cancer stem cell levels within a population. Therefore, it remains a question of whether cell surface GRP78 can be defined as a conventional cancer stem cell marker. Hypothetically, cancer stem cells do not divide frequently; they achieve self–renewal to maintain pluripotency-like or multipotency-like cancer stemness by undergoing asymmetric cell division, during which two offspring cells, each predisposed to different cell fates, are generated. One of the daughter cells retains cancer stemness, possessing the capability of unlimited cell proliferation without the fate of differentiation, senescence, or death. The other daughter cell is a committed progenitor that hypothetically divides frequently and symmetrically, contributing to the constitution of the tumor mass and volume^29,30,34–36^. The frequency of symmetric versus asymmetric cell division was not well characterized in cancer types other than breast cancers^30^ before our study. How cancer stem cells decide to undergo symmetric or asymmetric cell division remains unknown. Additionally, whether cancer stemness is maintained exclusively through asymmetric cell division and the time span for each cell cycle phase in asymmetric and symmetric cell divisions is also unknown. While harvesting cells for our asymmetric cell division assay, we observed some dividing cells during late telophase that remained attached to the cell culture plates after the first round of trypsinization. We also noticed that the BrdU-labeled cells are more resistant to trypsinization than the parental cells. This phenomenon of stress tolerance was also reported in multilineage-differentiating stress-enduring (Muse) cells^37^. Therefore, we trypsinized the cells twice for thorough harvesting of cells, and our modified experimental procedures could overcome the disadvantages of cell harvesting by mitotic shake-off within a short time period. This modification was beneficial because of the unknown time span for each cell cycle phase in symmetric and asymmetric division, as well as stronger attachment and trypsin resistance. The results of our asymmetric cell division assay demonstrate that cell surface GRP78 expression confers higher percentages of both symmetric and asymmetric cell division in OECM1 cells; a similar trend was observed in FaDu and BM2 cells but to a lesser extent. OECM1 and BM2 cells are known to proliferate, migrate, and invade much faster than FaDu cells^15^. Therefore, the overall result of this asymmetric cell division assay suggests that the expression level of cell surface GRP78 is proportional to the frequency of both asymmetric and symmetric cell division in the HNC cells of highly malignant phenotypes (such as OECM1), whereas less malignant HNC cells (such as FaDu) or HNC cells expressing lower levels of cell surface GRP78 (such as BM2) employ a mechanism in which cell surface GRP78 may not be the only involved molecule for cell fate determination, thereby resulting in a lower frequency of asymmetric cell division. Meanwhile, the percentages of cells undergoing asymmetric cell division (gate R4) in the total populations of OECM1, FaDu, and BM2 cells were below 5%, which is typical of the low cancer stem cell numbers within a cancer cell population. The percentage of asymmetric cell division in the total population of OECM1 cells was higher than that of FaDu and BM2 cells, indicating that highly malignant types of head and neck cancers possess a higher level of self-renewal achieved by asymmetric cell division than that of the less malignant types. The overall results of this assay suggest that cell surface GRP78 serves as the bridge directing self-renewal achieved by asymmetric and/or symmetric cell division from lower to higher efficiency during tumorigenesis or carcinogenesis.

Because cancer stemness is proposed to be a pluripotency-like phenotype, and resistance to chemo- and radiotherapy is suggested to be caused by the hypothetical low-dividing frequency of cancer stem cells, we examined the relationship of cell population heterogeneity based on the cell surface levels of GRP78 and Progranulin in OECM1, FaDu, and BM2 cells to cell cycle distributions and the relative expression of genes that are commonly used as markers for cancer stemness and developmental pluripotency. In OECM1 cells, all 5 markers (Nanog, Oct4, Sox2, TERF-1 and PRDM14) were up-regulated in the GRP78^Hi^PGN^−^ group, and the percentage of GRP78^Hi^PGN^−^ cells in the G2/M phase was also significantly higher. This trend of cell cycle distribution and stemness marker expression was also observed in FaDu cells, except for PRDM14, which was not expressed at significantly higher levels in FaDu cells expressing cell surface GRP78. This parallel between G2/M phase enrichment and up-regulated levels of cancer stemness echoes a recent report that the G2/M phase favors developmental pluripotency; in this report, by gene silencing or chemical treatment, prolonged S and G2 phases can prevent pluripotency state dissolution in human embryonic stem cells, whereas an extended G1 phase may lead to differentiation but does not inhibit pluripotency markers when bFGF or TGF-β is removed^27^. Furthermore, HNC cells of cell surface GRP78^Hi^ display stronger *in vivo* tumorigenesis than cell surface GRP78^Lo^ cells^16^. The malignancy levels (such as *in vitro* and *in vivo* tumorigenesis rate and invasion efficiency) of OECM1 cells are higher than those of FaDu cells, suggesting that highly malignant head and neck cancers possess higher levels of cancer stemness than less malignant ones. In BM2 cell line which expresses lower level of cell surface GRP78, only part of the 5 stemness markers showed statistically significant results, indicating that the expression levels of cell surface GRP78 are very important to the accomplished levels/phases of cancer stemness. Comparing the GRP78^Hi^PGN^-^ groups among OECM1, FaDu, and BM2 cells, it is very interesting that the frequency of cells in G1 phase is inversely proportional to the cell surface levels of GRP78. Therefore, the levels of cell surface GRP78 seem to be critical criteria for HNC cells to progress toward G2/M phase. In our study, GRP78 silenced did not affect cell cycle distribution in OECM1 cells. Since population heterogeneity due to different GRP78 levels still existed after GRP78 knockdown was achieved, the difference in the cell cycle distribution among cells of higher versus lower levels of GRP78 could not be exposed without cell sorting. This reflects the importance of population heterogeneity (determined by the cell surface levels of GRP78 and Progranulin in our model) to cell cycle distribution in HNC cells. It also suggests that cell surface localization of GRP78 is a critical process for maintaining cancer stemness or initiating cell reprogramming. Reconciling the pervious and our findings, cancer stem cells appear to be active in growth, division, self-renewal, and invasion, rather than dividing infrequently and remaining dormant.

Although the high percentage of OECM1, FaDu, and BM2 cells expressing cell surface GRP78 does not correspond to the characteristic of low percentage in a population of a conventional cancer stem cell marker (such as CD24), OECM1, FaDu, and BM2 cells with cell surface expression of GRP78 exhibit multiple characteristics of cancer stemness. For example, the cell surface GRP78^Hi^ cells displayed higher frequency of asymmetric and symmetric cell division, G2/M phase enrichment, and up-regulated expression of stemness-associated markers. Although PRDM14 was exclusively up-regulated in the GRP78^Hi^PGN^−^ group of OECM1 cells, this result was not observed in FaDu cells. It will be important to investigate whether the development or the maintenance of cancer stemness is also a multi-staged process, like somatic cell reprogramming^38^, and whether cell surface GRP78 and PRDM14 can promote cancer stemness to a fully developed state. Apoptosis and down-regulated expression of the five stemness-associated markers were observed in OECM1, FaDu, and BM2 cells with Progranulin chaperoned by GRP78 on the cell surface. Moreover, Progranulin-expressing cells performed only symmetric, but not asymmetric, cell division. Because Progranulin is involved in differentiation during embryogenesis, it is possible that head and neck cancer cells periodically activate apoptosis mechanisms to discard Progranulin-expressing cells for the maintenance of cancer stemness. Another possible mechanism is that symmetric cell division is highly attachment- or orientation-dependent, but asymmetric cell division is not. Therefore, once cells are detached from the cell culture plates, Progranulin-expressing cells cannot sustain their regular cell fate determination and quickly proceed to apoptosis, which indirectly indicates that Progranulin is a specific regulator of symmetric cell division. Collectively, we conclude that GRP78 is a chaperone possessing a deterministic role in maintaining cancer stemness. Cell surface expression of GRP78 promotes cancer stemness in HNC cells, but it can also drive FaDu and OECM1 cells away from cancer stemness via chaperoning Progranulin to the PM compartment.

## Materials and Methods

### Cancer Cell lines and Culture

The five human head and neck cancer cell (HNC) lines [Detroit (ATCC CCL-138), NPC076, BM2^39^, OECM1^40^, and FaDu (ATCC HTB-43)] used in this project were cultured in their respective medium base with 7% fetal bovine serum (Biological Industries, Cromwell, CT, USA) and 1% antibiotics (Corning, Corning, NY, USA). The medium base for Detroit and Fadu is MEM, that for OECM1 and BM2 RPMI1640 and NPC076 DMEM.

### Flow Cytometry Analysis and Fluorescence Activated Cell Sorting (FACS)

To analyze expression levels of cell surface GRP78 and to sort for groups of cells expressing different levels of GRP78 and Progranulin, HNC cells were incubated with GRP78 and Progranulin antibodies (Santa Cruz, Dallas, Texas, USA), or the corresponding isotype control immunoglobulins (JacksonImmunoResearch, Newmarket, Suffolk, UK), followed by staining with allophycocyanin (APC)- and peridinin chlorophyll protein (PerCP)-conjugated secondary antibodies (JacksonImmunoResearch). Propidium iodide and FITC Annexin V (Invitrogen, Waltham, MA, USA) were used to gate out dead and early apoptotic cells. Fluorescence signals were acquired using a FacsCalibur^TM^ flow cytometer (BD Biosciences, San Jose, CA, USA). FACS experiments were performed using a FacsAria^TM^ cell sorter (BD Biosciences). The overlay histograms were constructed using CellQuest Pro^TM^ Version 6.0 (BD Biosciences). The cell cycle histograms of the five sorted groups were plotted using the software ModFit LT^TM^ (Verity Software House, Topsham, Maine, USA).

### Fractionation of Plasma Membrane Proteins and Extraction of Total Cell Lysates

Fractionation of plasma membrane proteins was performed using Qproteome^TM^ Plasma Membrane Protein Kit (QIAGEN, Valencia, CA, USA) according to the manufacturer’s instructions. The non-plasma membrane (non-PM) fraction was constituted of the cytosolic fraction after plasma membrane isolation mixed with the supernatant extracted from the pellets left from plasma membrane fractionations. For extracting total cell lysates, cell pellets were harvested by trypsinization and homogenized in RIPA buffer (Sigma-Aldrich, St. Louis, MO, USA) supplemented with cocktails of protease inhibitors (QIAGEN) and phosphatase inhibitors (Roche, Werk Penzberg, Germany) before centrifugation at 12000 × g for 15 minutes at 4 °C. Total cell lysates, PM and non-PM proteins were quantified using a Bradford assay (Bio-Rad, Hercules, CA, USA).

### Co-immunoprecipitation and Western Blot Analysis

The co-immunoprecipitations and western blot analyses were performed similarly as previously described^41^. Antibodies recognizing GRP78, Progranulin, GRXCR2, Serpin H1, BBS4, TCP-1 theta, HRG, BLOC1S2, and PRMT1 were purchased from Santa Cruz. Briefly, equal amounts of PM or non-PM proteins were incubated with the anti-GRP78 antibody or the isotype control immunoglobulin at 4 °C overnight. The zwitterionic detergent CHAPS (2%) was included in all of the immunoprecipitation reactions. Dynabeads^®^ protein G magnetic beads (Thermo Fisher Scientific, Waltham, MA, USA) or protein A/G agarose beads (Calbiochem^®^, Temecula, CA, USA) were added to the non-PM and PM samples, respectively, and were rotated at 4 °C overnight. To verify successful GRP78 immunoprecipitation and the following interactome candidates by western blot analysis, one-sixth of the volume of the beads carrying the immunoprecipitants from the PMs were aliquoted, washed twice with PBS, and then heated in sample buffer for 10 minutes at 90 °C, followed by vigorous vortexing. The immunoprecipitated proteins were separated by SDS-PAGE electrophoresis and then were transferred to nitrocellulose membranes. Protein bands in the blots were photographed using a UVP610 imaging system (Biocompare, Billerica, MA, USA) and were quantified using ImageJ software (National Institutes of Health, Bethesda, Maryland, USA). The nitrocellulose membranes were stripped in 0.5 M Tris buffer (pH 6.8) containing 10% SDS and 0.8% β-mercaptoethanol at 50 °C for 20 minutes before re-probing.

### Two-Dimensional Liquid Chromatography Mass Spectrometry (2D LC-MS/MS)

Our platform for 2D LC-MS/MS was modified from previously described protocols^26^. The immunoprecipitants captured by the beads were eluted with 0.1% trifluoroacetic acid/50% acetonitrile (Sigma-Aldrich), the beads were removed, and the solution was dried by speed vacuum. Dithiothreitol (Sigma-Aldrich) and iodoacetamide (Sigma-Aldrich) was added for reduction and alkylation, respectively. Then, sequencing grade trypsin (Promega, Madison, WI, USA) was added for in-solution digestion. The tryptic digests of the immunoprecipitants captured by the anti-GRP78 antibody and the isotype control immunoglobulin were labeled with formaldehyde-D_2_ (Isotec, Miamisburg, OH, USA) and formaldehyde (Isotec), respectively. The formaldehyde-D_2_- and formaldehyde-H_2_-labeled peptides were mixed in a ratio of 1:1 and then were subjected to a comprehensive 2D-SCX-RP-LC system (for the non-PM samples, Thermo Scientific Dionex, Bannockburn, IL, USA) or to a C18 column (for the PM samples, Acclaim PepMap RSLC, Thermo Scientific Dionex) for peptide separation. The effluents of the 2D-SCX-RP-LC system and the C18 column were analyzed using an LTQ-Orbitrap hybrid mass spectrometer (Thermo Electron, Bremen, Germany) and a Q Exactive mass spectrometer (Thermo Scientific) coupled with an Ultimate 3000 RSLC system (Dionex), respectively. Raw MS files were analyzed by Mascot (Version 2.2.2, Matrix Science Inc., Boston, MA, USA) using the database SwissProt 2010 (Geneva, Switzerland).

### Confocal Microscopy of Live Cells

Confocal microscopy was performed according to the LSM 780 user manual (Zeiss, Oberkochen, Germany) and the μ-slide 8 well user instruction (ibidi^®^, Martinsried, Germany). Briefly, parental OECM1 and FaDu cells were seeded in 8-well μ-slides one day before immunofluorescence staining was performed. The nuclei were stained with Hoechst 33342 (Bio-Rad). For the interactome candidates that are at the intracellular side of the plasma membrane, the live cells were incubated with the GRP78 antibody (Santa Cruz) and the APC conjugated secondary antibody (JacksonImmunoResearch), followed by fixation with 1% formaldehyde. Cell were then permeabilized with 0.5% Tween 20, followed by incubation with primary antibodies recognizing the interactome candidates and the appropriate PerCP-conjugated secondary antibody (JacksonImmunoResearch). For extracellular interactome candidates, live cells were not fixed nor permeabilized before or after antibody staining. PBS containing 5% FBS was added to the cells, and then the fluorescence images were acquired using an LSM780 confocal microscope (Zeiss).

### Asymmetric Cell Division Assay

The asymmetric cell division assay was modified from previously described protocols^29^. Briefly, BM2 and OECM1 cells were cultured in their growth media containing 1 μM BrdU (Sigma-Aldrich) for 14 days, and FaDu cells 11 days. Then, the BrdU was withdrawn for 24 and 48 hours before the cells were harvested by trypsinization and immunostained with Progranulin and GRP78 antibodies. These cells were then fixed with 4% paraformaldehyde before permeabilizing with 0.5% NP40 and staining with a FITC-conjugated BrdU antibody (Santa Cruz) before flow cytometry analysis.

### Reverse Transcription and Quantitative Real-Time PCR Analysis

After total RNA was extracted using TRIzol (Gibco, Waltham, MA, USA) and treated with DNase I (Invitrogen, Waltham, MA, USA), reverse transcription was performed using MMLV Reverse Transcriptase (Invitrogen). Quantitative real-time PCR was performed using iQ^TM^ SYBR^®^ Green Supermix (Bio-Rad) on a MiniOpticon^TM^ System in conjugation with CFX Manager^TM^ software (Bio-Rad). The primer sequences of Nanog, Oct4, Sox2, TERF-1, and PRDM14 were described previously^42–45^. For each sample, the mRNA levels of each gene were normalized to that of GAPDH and were calculated as the relative quantification cycle (ΔΔCq) relative to the Igs group as the control sample.

### GRP78 Knockdown

For silencing GRP78, OECM1 cells were transfected with a plasmid carrying a small hairpin RNA (shRNA) complementary to GRP78 mRNA^15^ or a scramble shRNA^15^ using jetPRIME^®^ reagent (Polyplus-transfection, New York, NY, USA) according to the suggested protocol in the user’s booklet. Briefly, 5 μg of plasmids and 10 μL of jetPRIME^®^ reagent were applied to 10^6^ cells. After incubating for 4 hours, the transfection mixtures were removed and cells were recovered in the regular growth medium for 12 hours before harvesting by trypsinization.

### Statistical Analysis

All quantitative data are presented as mean ± SEM. The statistical analyses for the data consisted of 5 groups were performed with one-way ANOVA and then followed with Newman-Keuls multiple comparison tests using GraphPad Prism 6 (GraphPad Software, La Jolla, CA, USA), and that for the data consisted of 2 groups unpaired t test using GraphPad Prism 6. The numbers of independent experiments and biological replicates for each study were described in the respective figure legends. Significant levels were determined as *p < 0.1, **p < 0.05, ***p < 0.01, ****p < 0.001, and *****p < 0.0001. NS is the abbreviation of not significant.

## Electronic supplementary material

Supplementary Information
