# Supplementary material for: The Endogenous GRP78 Interactome in Human Head and Neck Cancers: A Deterministic Role of Cell Surface GRP78 in Cancer Stemness
Source: Sci Rep. 2018 Jan 11;8:536. doi: 10.1038/s41598-017-14604-5 (PMC5765009; doi:10.1038/s41598-017-14604-5)
Supplement: Supplementary file 1 — Supplementary Information [file 41598_2017_14604_MOESM1_ESM.pdf]

The Endogenous GRP78 Interactome in Human Head and Neck Cancers: A Deterministic Role of Cell Surface GRP78 in Cancer Stemness

Hsin-Ying Chen, Joseph Tung-Chieh Chang, Kun-Yi Chien, Yun-Shien Lee, Guo-Rung You, and Ann-Joy Cheng

Supplemental Figures and Tables

Fig S1

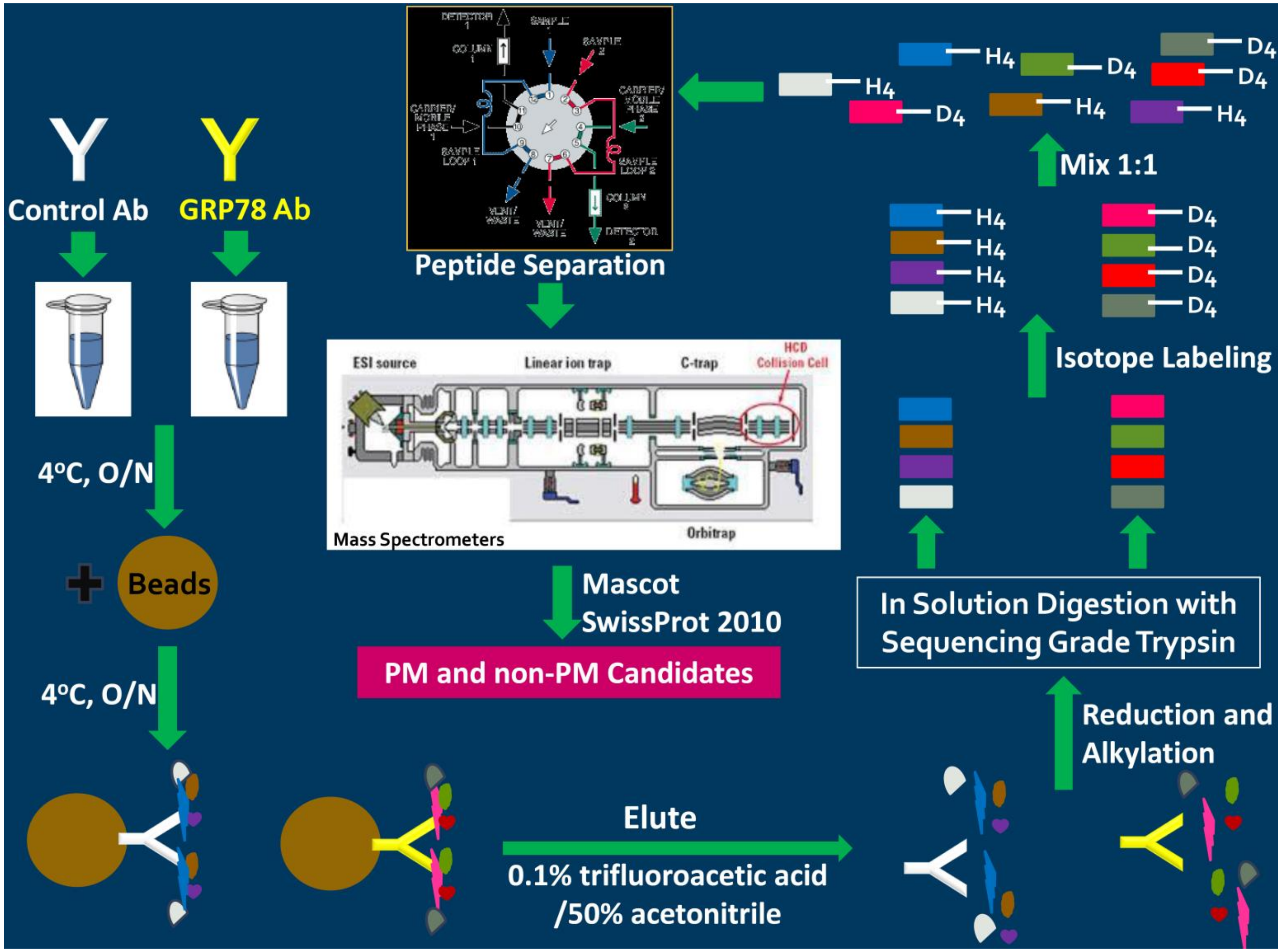

Fig S1 The Graphical procedures of co-immunoprecipitation by a GRP78 antibody and an isotype control immunoglobulin followed by 2D LC-MS/MS and Mascot Database-Matching Algorithm for detection of GRP78 interactome.

Fig S2

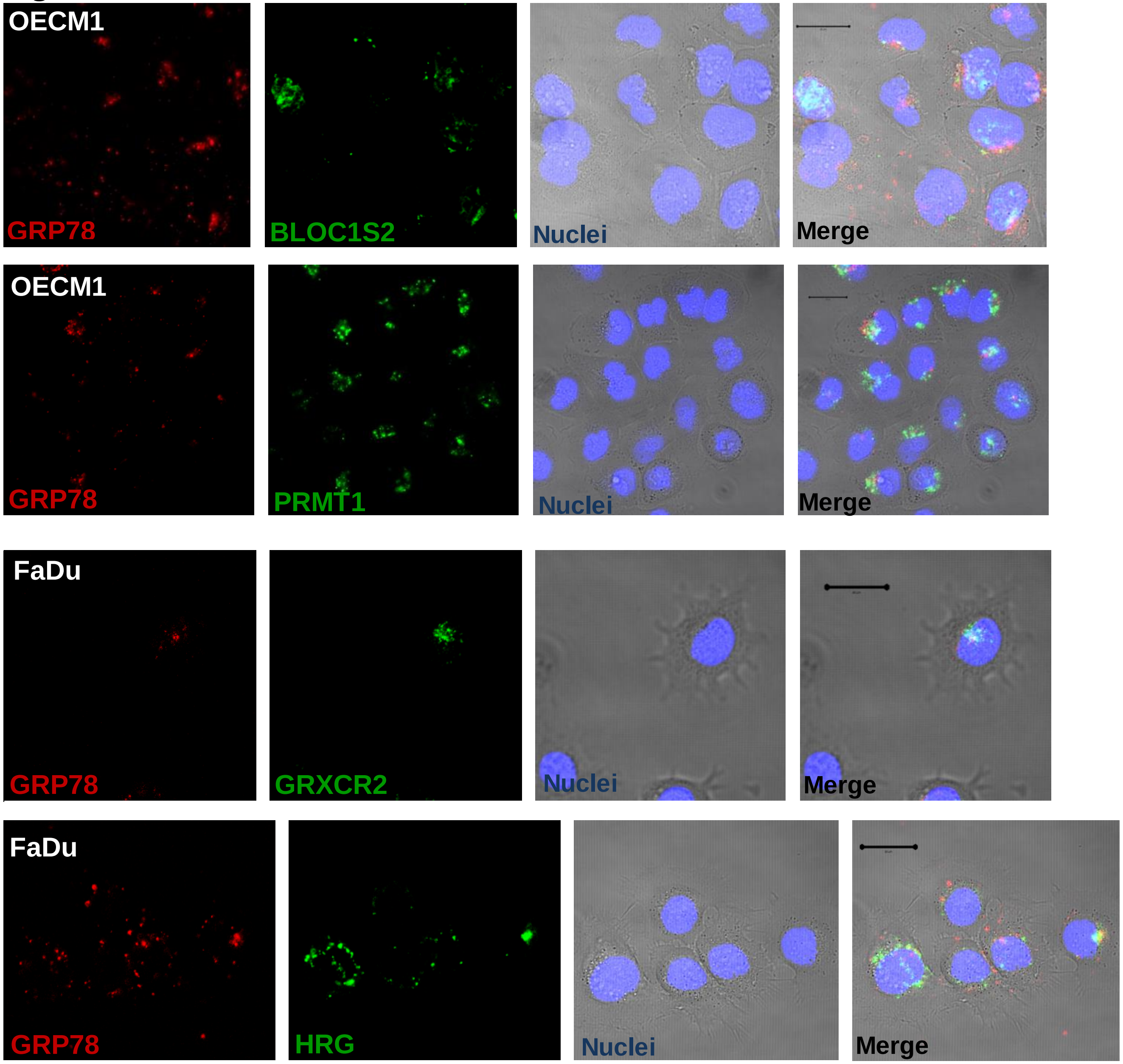
OECM1
GRP78
BLOC1S2
Nuclei
Merge
OECM1
GRP78
PRMT1
Nuclei
Merge
FaDu
GRP78
GRXCR2
Nuclei
Merge
FaDu
GRP78
HRG
Nuclei
Merge

Fig S2 Verification of the GRP78 interactome candidates in the PM compartment of OECM-1 and FaDu cells by confocal microscopy.

Only partial overlap between the fluorescence staining of BLOC1S2/GRP78, PRMT1/GRP78, GRXCR2/GRP78, and HRG/GRP78 was observed. The red immunostaining in the images represents GRP78, and the green immunostaining represents the interactome candidates. The nuclei were stained with Hoechst 33342. BLOC1S2, PRMT1, GRXCR2, and HRG are the interactome candidates on the cell surface. The fluorescence images were acquired using a 63X oil lens on an LSM780 confocal microscope. Scale bars = 20 μm.

Fig S3

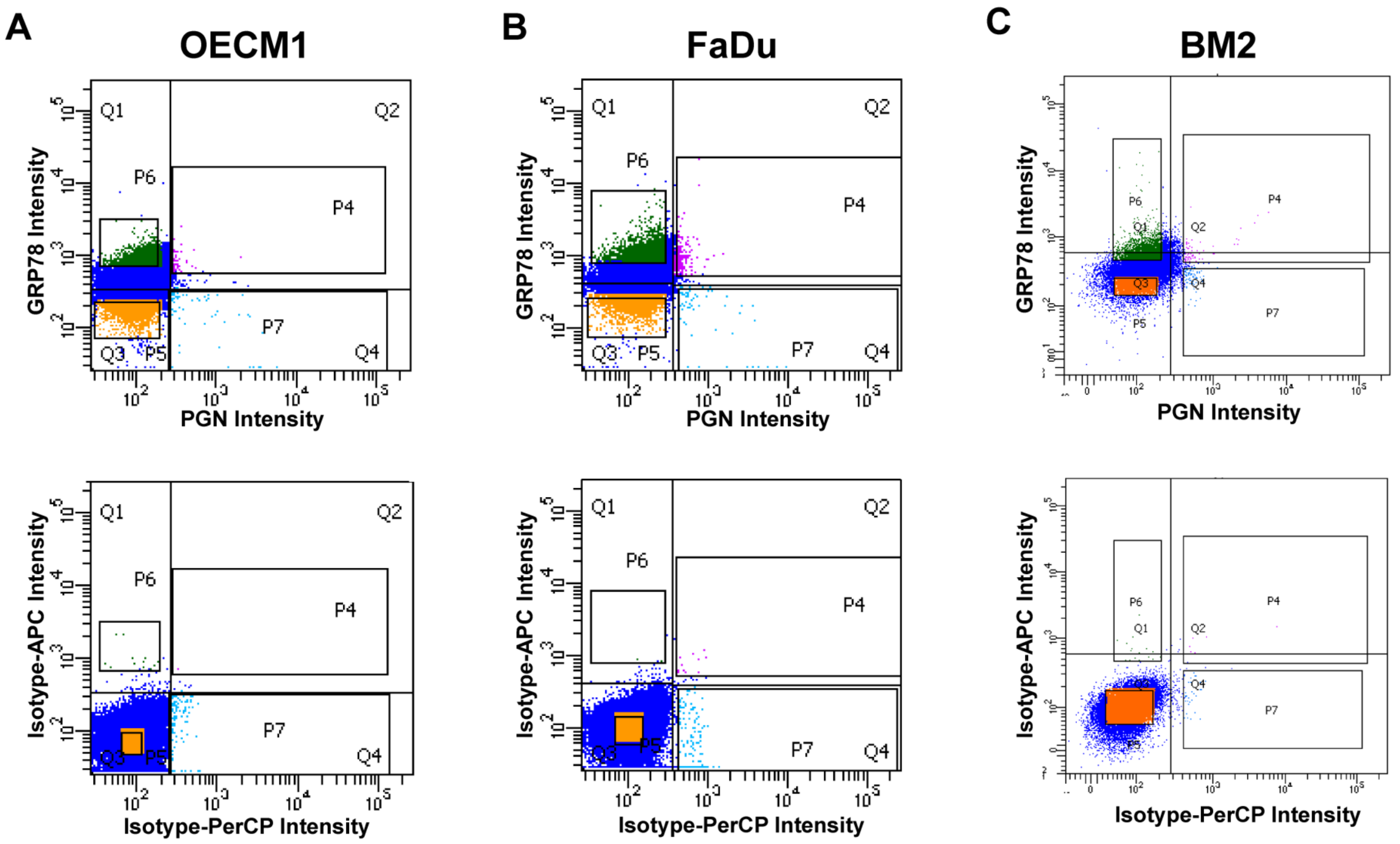

Fig S3 Gating scheme for fluorescence-activated cell sorting.

(A, B and C, upper plots) The entire cell populations of OECM1, FaDu and BM2 cells were sorted into four groups according to the cell surface levels of GRP78 and Progranulin. P6: $GRP78^{Hi}PGN^{-}$ group; P4: $GRP78^{Hi}PGN^{+}$ group; P7: $GRP78^{Lo}PGN^{+}$ group; P5: $GRP78^{Lo}PGN^{-}$ group. (A, B and C, lower plots) P5 (the Igs group) served as the control group. The Igs group consisted of cells incubated with the isotype control immunoglobulins corresponding to the antibodies recognizing GRP78 and Progranulin; this group represents the sham sorted total population.

Fig S4

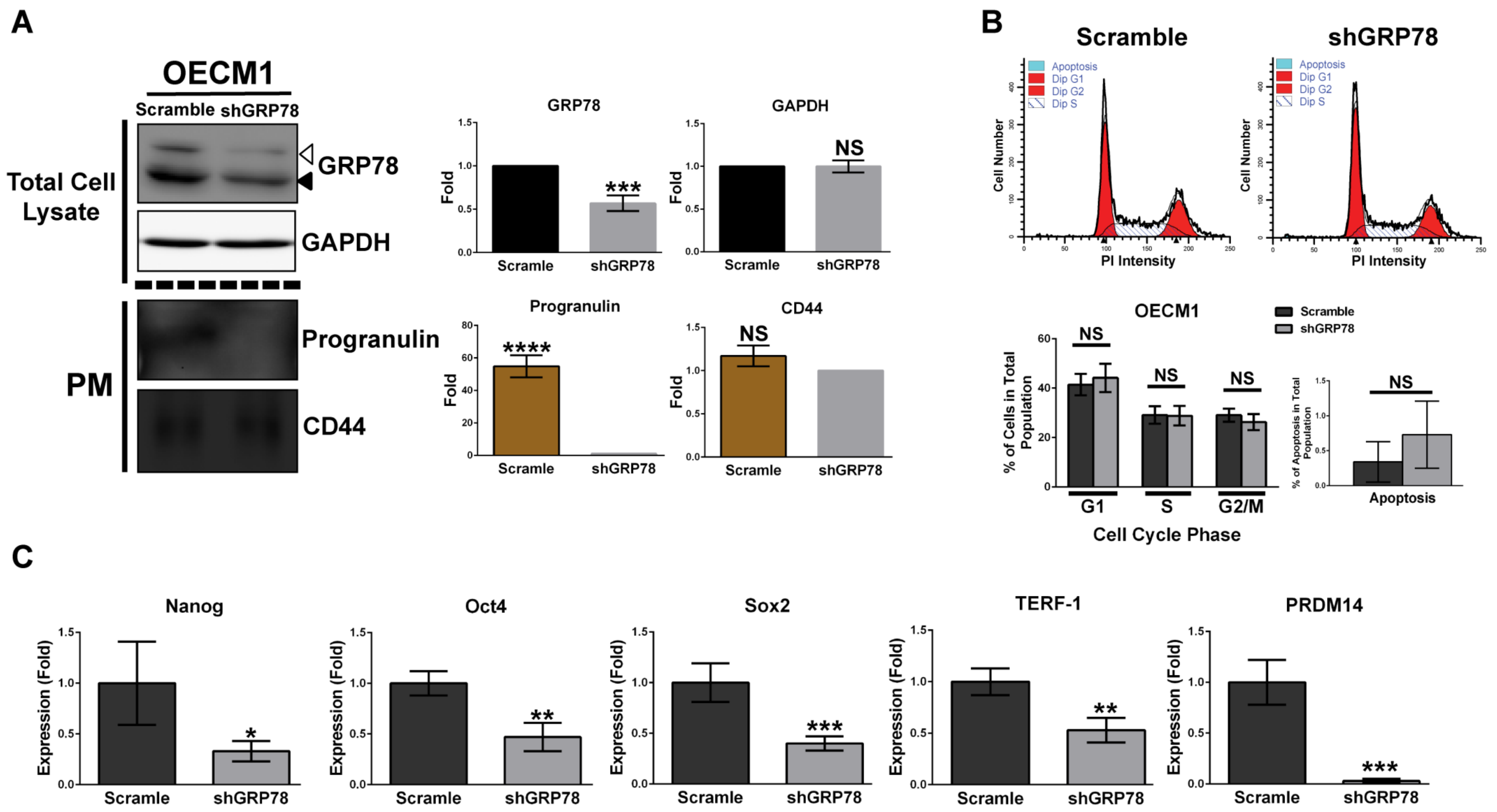

Fig S4 GRP78 silencing confirms the role of GRP78 as a deterministic chaperone in cancer stemness.

(A) After GRP78 knockdown by transfection with a small hairpin RNA complementary to GRP78 mRNA, Progranulin cannot express on the cell surface of OECM1 cells. The bar graphs in the right side were the mean ± SEM quantified from 3 Western Blots of 3 independent knockdown experiments with OECM1 cells. Both the upper (cytosolic form, pointed by an open arrow head) and lower (membranous form, pointed by a solid arrow head) bands of GRP78 were quantified together as the

expression levels of GRP78. (B) GRP78 silencing does not affect the cell cycle distribution. The bar graphs in the lower side were the mean ± SEM of 3 replicates from 3 independent knockdown experiments. Protein bands of all of the blots were quantified using ImageJ software. Full-length blots are presented in Supplement II Figure S8. (C) All of the 5 stemness associated markers were significantly reduced in GRP78 silenced OECM1 cells. Data in each bar graph were the mean ± SEM of five replicates from the two independent knockdown experiments of OECM1 cells; $*p < 0.1$, $**p < 0.05$, $***p < 0.01$, and $****p < 0.001$; NS = not significant.

Table S1

The top 25 ranked interactome candidates of GRP78 in the non-plasm membrane compartment of OECM1 cells

Accession No.	Gene Name	Protein Name	No. of Matched Proteins	No. of Identified Peptides	No. of Unique Peptides	Score	Coverage %	D4/H4 Ratio
P23246	SFPQ	Splicing factor, proline- and glutamine-rich	1	12	6	373.93	19.94	317.49
Q08211	DHX9	ATP-dependent RNA helicase A	1	18	14	1211.31	22.05	272.12
P07910	HNRNPC	Heterogeneous nuclear ribonucleoproteins C1/C2	2	14	10	507.22	35.29	245.32
O15372	EIF3H	Eukaryotic translation initiation factor 3 subunit H	1	3	1	216.72	13.92	152.30
P14866	HNRNPL	Heterogeneous nuclear ribonucleoprotein L	1	11	7	1354.67	38.37	136.97
P26599	PTBP1	Polypyrimidine tract-binding protein 1	1	12	7	593.20	37.48	125.67
P09651	HNRNPA1	Heterogeneous nuclear ribonucleoprotein A1	3	16	11	910.89	41.13	92.47
P67809	YBX1	Nuclease-sensitive element-binding protein 1	2	10	6	684.55	43.52	90.27
Q14103	HNRNPD	Heterogeneous nuclear ribonucleoprotein D0	2	4	3	151.05	16.06	81.73
Q02543	RPL18A	60S ribosomal protein L18a	1	4	1	58.66	18.18	73.86
P18124	RPL7	60S ribosomal protein L7	1	14	6	334.82	50.00	71.78
P32969	RPL9	60S ribosomal protein L9	1	4	3	264.44	38.54	70.70
P62277	RPS13	40S ribosomal protein S13	1	7	6	248.81	45.03	61.54
P35637	FUS	RNA-binding protein FUS	2	10	7	613.89	24.71	60.23
P08708	RPS17	40S ribosomal protein S17	1	2	1	110.89	32.59	57.69
P46778	RPL21	60S ribosomal protein L21	1	2	1	163.66	13.75	52.85
P61247	RPS3A	40S ribosomal protein S3a	1	10	6	281.93	36.74	50.99
P15880	RPS2	40S ribosomal protein S2	1	9	5	404.21	34.81	50.30
P61978	HNRNPK	Heterogeneous nuclear ribonucleoprotein K	1	10	7	351.61	31.53	43.58
Q8TCJ2	STT3B	Dolichyl-diphosphooligosaccharide--protein glycosyltransferase subunit STT3B	1	3	1	56.95	7.26	40.68
Q9UQE7	SMC3	Structural maintenance of chromosomes protein 3	1	9	3	289.03	10.60	36.62
P05387	RPLP2	60S acidic ribosomal protein P2	1	5	4	359.43	73.04	35.26
P39023	RPL3	60S ribosomal protein L3	1	6	5	286.21	22.83	34.98
O43809	NUDT21	Cleavage and polyadenylation specificity factor subunit 5	1	5	4	267.37	29.52	32.89
P62249	RPS16	40S ribosomal protein S16	1	6	3	117.53	32.19	32.72

Table S2

The top 25 ranked interactome candidates of GRP78 in the non-plasm membrane compartment of FaDu cells

Accession No.	Gene Name	Protein Name	No. of Matched Proteins	No. of Identified Peptides	No. of Unique Peptides	Score	Coverage %	D4/H4 Ratio
P25205	MCM3	DNA replication licensing factor MCM3	1	25	25	1601.25	46.41	174.83
P0C0L4	C4A	Complement C4-A	2	3	3	237.54	2.47	95.15
Q6DD88	ATL3	Atlastin-3	1	10	10	943.51	31.61	73.62
Q9HCC0	MCCC2	Methylcrotonoyl-CoA carboxylase beta chain, mitochondrial	1	16	16	1716.52	42.98	65.95
Q96RQ3	MCCC1	Methylcrotonoyl-CoA carboxylase subunit alpha, mitochondrial	1	12	12	809.85	22.90	38.21
P33993	MCM7	DNA replication licensing factor MCM7	1	21	21	1404.37	44.51	33.93
Q00610	CLTC	Clathrin heavy chain 1	1	8	8	481.89	6.75	30.19
O00165	HAX1	HCLS1-associated protein X-1	1	6	6	237.17	40.50	30.09
Q01469	FABP5	Fatty acid-binding protein, epidermal	1	1	1	54.76	6.67	26.34
Q8IZ83	ALDH16A1	Aldehyde dehydrogenase family 16 member A1	1	2	2	156.09	2.99	22.28
P50416	CPT1A	Carnitine O-palmitoyltransferase 1, liver isoform	1	3	3	133.00	7.50	22.13
P33991	MCM4	DNA replication licensing factor MCM4	1	17	17	1252.34	32.33	21.55
O75955	FLOT1	Flotillin-1	1	1	1	115.31	3.04	21.07
Q15075	EEA1	Early endosome antigen 1	1	1	1	45.51	1.13	19.70
P49736	MCM2	DNA replication licensing factor MCM2	1	3	3	217.75	4.09	18.76
Q14254	FLOT2	Flotillin-2	1	4	4	156.90	14.95	17.65
Q9H078	CLPB	Caseinolytic peptidase B protein homolog	1	10	10	500.86	20.08	16.60
Q14204	DYNC1H1	Cytoplasmic dynein 1 heavy chain 1	1	3	3	118.64	1.49	14.08
P21333	FLNA	Filamin-A	1	25	25	1419.00	15.00	14.00
P39023	RPL3	60S ribosomal protein L3	1	1	1	123.94	2.98	13.80
P04083	ANXA1	Annexin A1	1	2	2	318.37	9.83	12.43
P19338	NCL	Nucleolin	1	3	3	105.36	6.90	11.68
P61313	RPL15	60S ribosomal protein L15	1	2	2	74.79	10.29	10.87
P27708	CAD	CAD protein	1	6	6	300.89	3.10	10.49
Q5RKV6	EXOSC6	Exosome complex exonuclease MTR3	1	1	1	164.45	5.88	10.42

Table S3

The detected interactome candidates of GRP78 in the plasm membrane compartment of OECM1 and FaDu cells

Cell Line	Accession No.	Gene Name	Protein Name	No. of Matched Proteins	No. of Identified Peptides	No. of Unique Peptides	Score	Coverage %	D4/H4 Ratio	Value of Deuterium Labeling Only
OECM1	P11021	HSPA5	78 kDa glucose-regulated protein	1	6	4	108.55	13.61	14.085	-
	P28799	GRN	Granulins	1	2	2	134.43	4.89	1.927	-
	Q6QNY1	BLOC1S2	Biogenesis of lysosome-related organelles complex 1 subunit 2	1	2	1	32.61	28.17	10.227	-
	P0C0L4	C4A	Complement C4-A	2	1	1	83.50	0.86	-	2.907x10^{7}
	Q99873	PRMT1	Protein arginine N-methyltransferase 1	2	1	1	49.24	3.32		1.624x10^{6}
	Q9NR22	PRMT8	Protein arginine N-methyltransferase 8	2	3	1	49.24	10.41	-	-
	Q8WZ42	TITIN	Titin	1	79	1	35.31	4.51	-	-
	Q9GZR1	SENP6	Sentrin-specific protease 6	1	3	1	33.01	6.29	-	-
	O14979	HNRNPDL	Heterogeneous nuclear ribonucleoprotein D-like	1	2	1	35.51	9.76	-	-
	Q9H8K7	C10orf88	Uncharacterized protein C10orf88	6	5	1	26.12	20.67	-	-
	O60716	CTNND1	Catenin delta-1	1	3	1	0.00	5.48	-	-
FaDu	A6NFK2	GRXCR2	Glutaredoxin domain-containing cysteine-rich protein 2	1	1	1	42.92	3.63	57.050	-
	P50454	SERPINH1	Serpin H1	1	1	1	51.78	2.63	4.472	-
	P28799	GRN	Granulins	1	3	3	264.42	7.93	1.039	-
	Q5M775	SPECC1	Cytospin-B	2	1	1	196.83	0.66	*3.376	-
	P50990	CCT8	T-complex protein 1 subunit theta	1	1	1	53.79	1.09	-	6.625x10^{6}
	P04196	HRG	Histidine-rich glycoprotein	1	2	1	76.24	3.05	-	4.345x10^{6}
	Q96RK4	BBS4	Bardet-Biedl syndrome 4 protein	2	1	1	43.30	1.16	-	2.556x10^{7}
	P0C0L4	C4A	Complement C4-A	2	1	1	263.25	0.86	-	1.009x10^{7}
	Q494V2	CCDC37	Coiled-coil domain-containing protein 37	1	1	1	40.78	0.82	-	8.472x10^{6}
	Q8N8M0	C7orf52	Putative N-acetyltransferase C7orf52	1	1	1	42.27	2.44	*100	*6.225x10^{7}
	P51153	RAB13	Ras-related protein Rab-13	2	1	1	31.92	2.96	4.638	-
	O75762	TRPA1	Transient receptor potential cation channel subfamily A member 1	8	2	1	31.98	0.89	56.108	-
	Q6ISU1	PTCRA	Pre T-cell antigen receptor alpha	5	1	1	32.78	1.78	*25.196	-
	Q13823	GNL2	Nucleolar GTP-binding protein 2	1	1	1	45.18	1.92	-	-

	Q8N442	GUF1	GTP-binding protein GUF1 homolog	1	1	1	43.25	1.05	-	-
	P03372	ESR1	Estrogen receptor	1	1	1	43.04	1.51	-	-
	P54577	YARS	Tyrosine--tRNA ligase, cytoplasmic	1	1	1	30.07	2.08	-	-
	P61296	HAND2	Heart- and neural crest derivatives-expressed protein 2	1	1	1	21.08	4.15		

Table S3 The detected interactome candidates of GRP78 in the plasm membrane compartment of OECM1 and FaDu cells.

Except for immunoglobulin-related proteins, bovine serum albumin, and keratin-related proteins, all of the detected interactome candidates are listed. The candidates with only deuterium labeling have no D4/H4 ratios because the signals of hydrogen labeling are not detectable. This occurs when no corresponding ions are generated from the immunoprecipitants pulled-down by the isotype control immunoglobulin relative to that by the anti-GRP78 antibody, which represents the interactome candidates associating with cell surface GRP78 with high specificities.

* More than half of the identified peptides of this protein candidate have D4/H4 ratios and/or values of deuterium labeling only. Therefore, the averages of the detected D4/H4 ratios and/or values of deuterium labeling were calculated and are shown in this column.

Fig S5 part 1

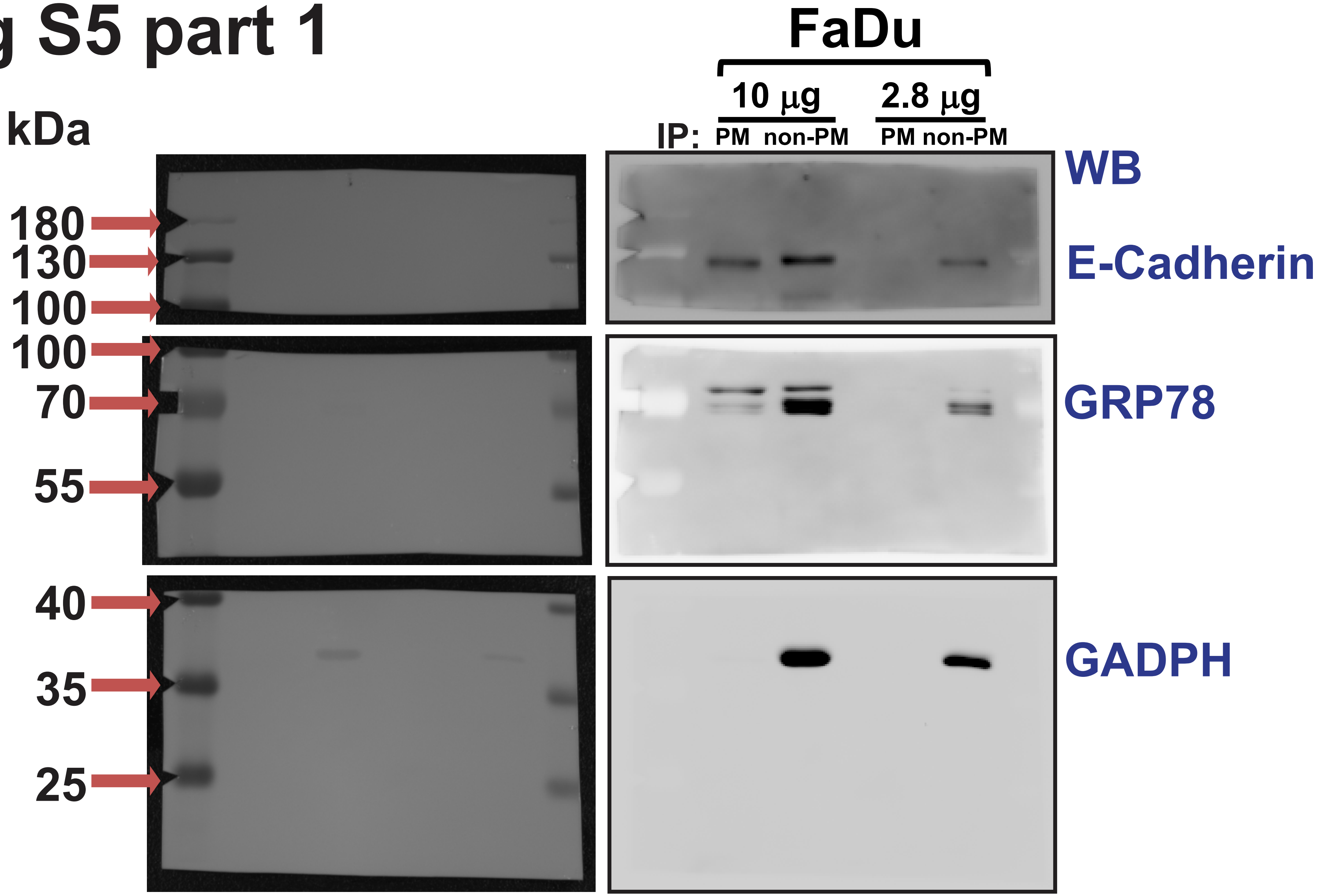

These three blots were cut from one membrane and are the uncropped images of the left blots in Fig 2A.

Fig S5 part 2

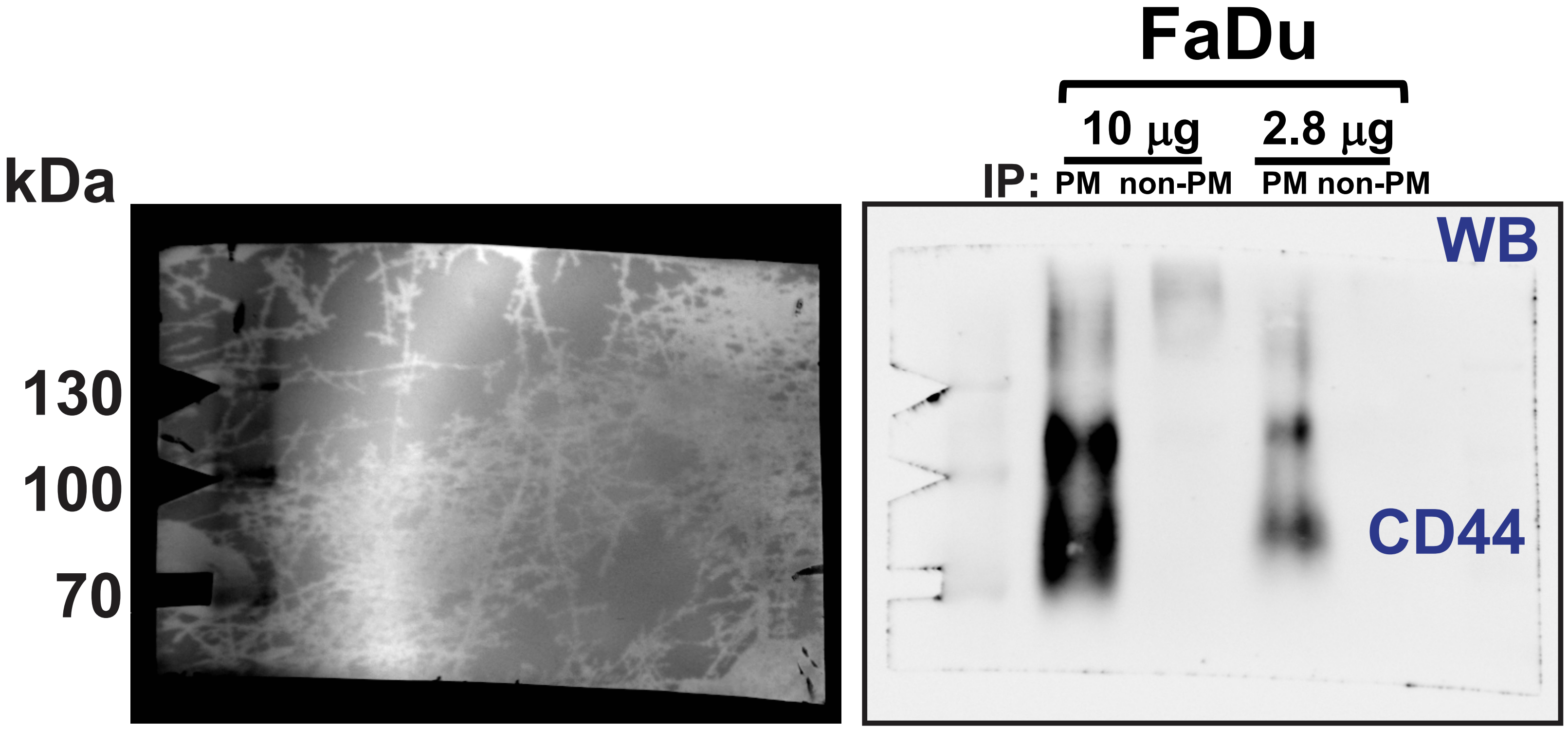

Size of CD44 = 82 kDa

This blot is the uncropped image of one of the left blots in Fig 2A.

Fig S5 part 3

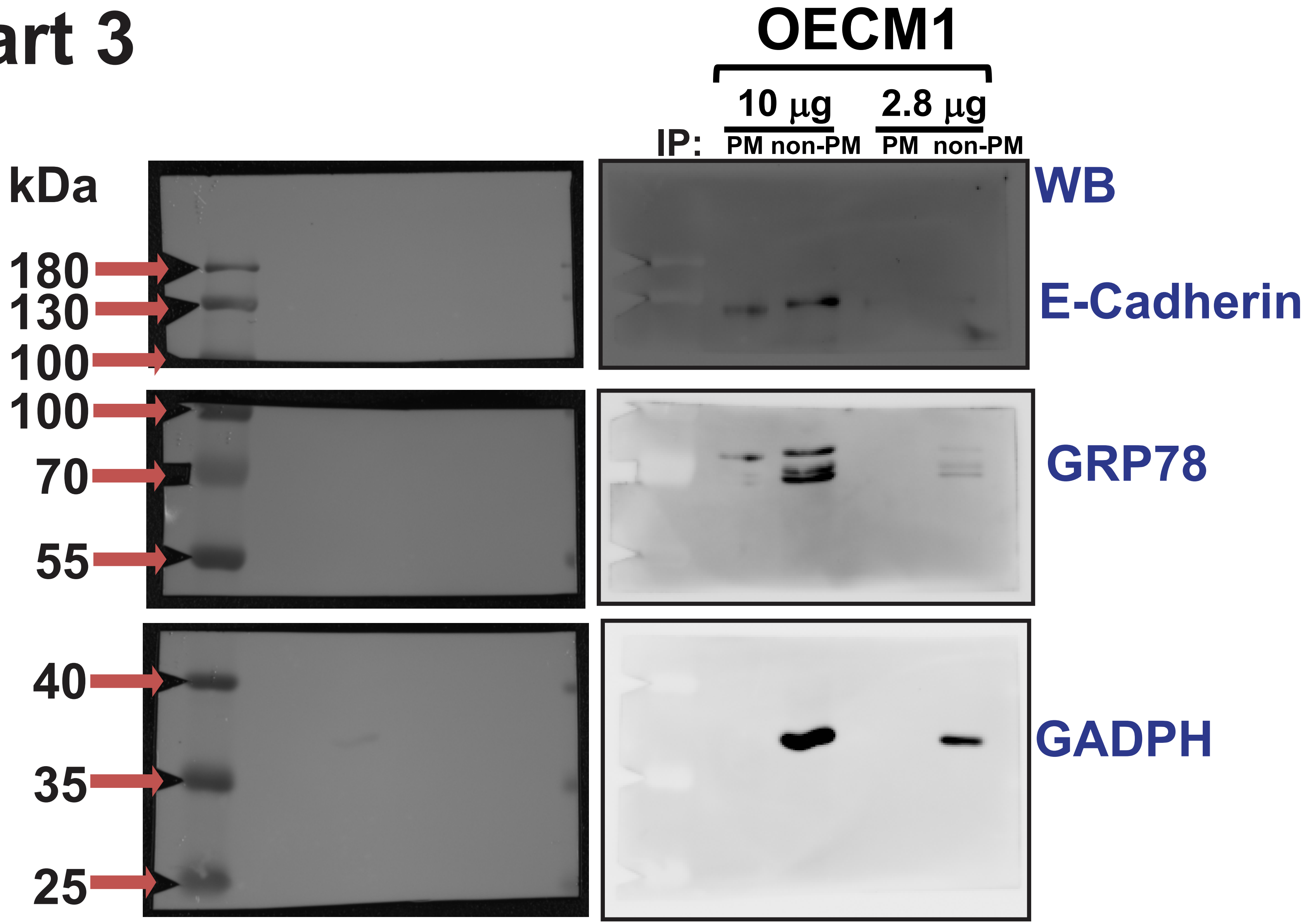

These three blots were cut from one membrane and are the uncropped images of the right blots in Fig 2A.

Fig S5 part 4

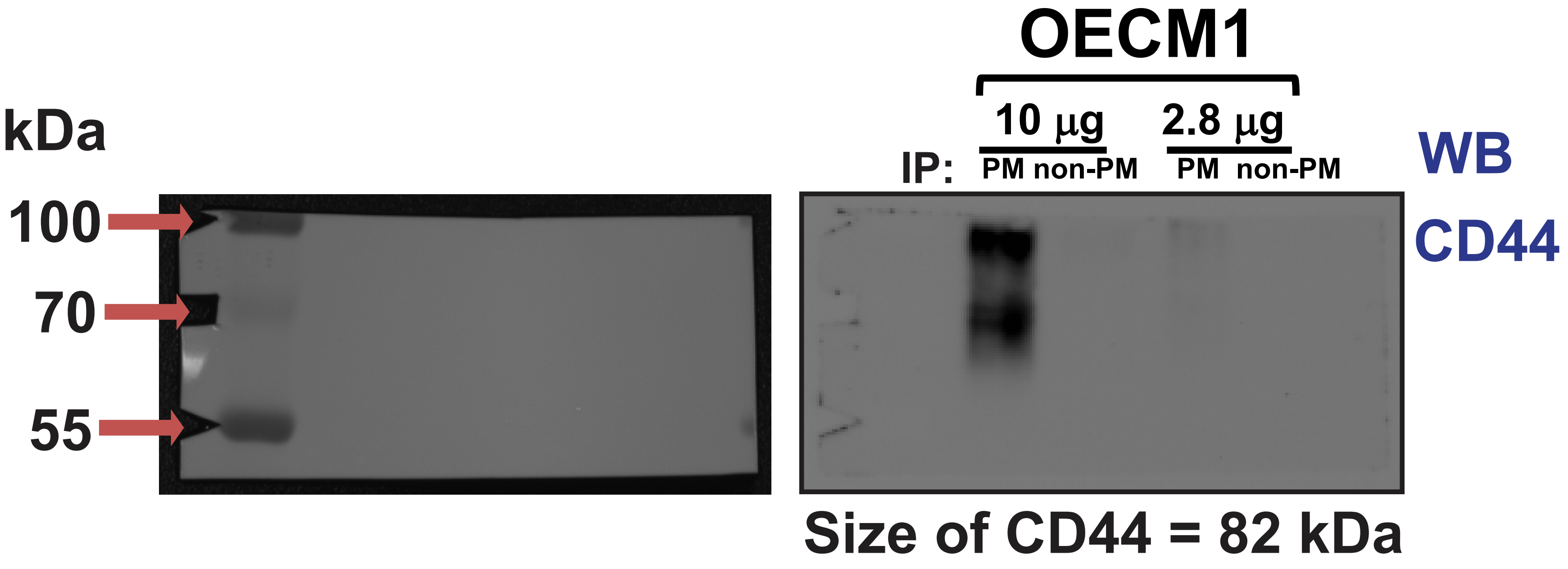

This blot is the uncropped image of one of the right blots in Fig 2A.

Fig S6 part 1

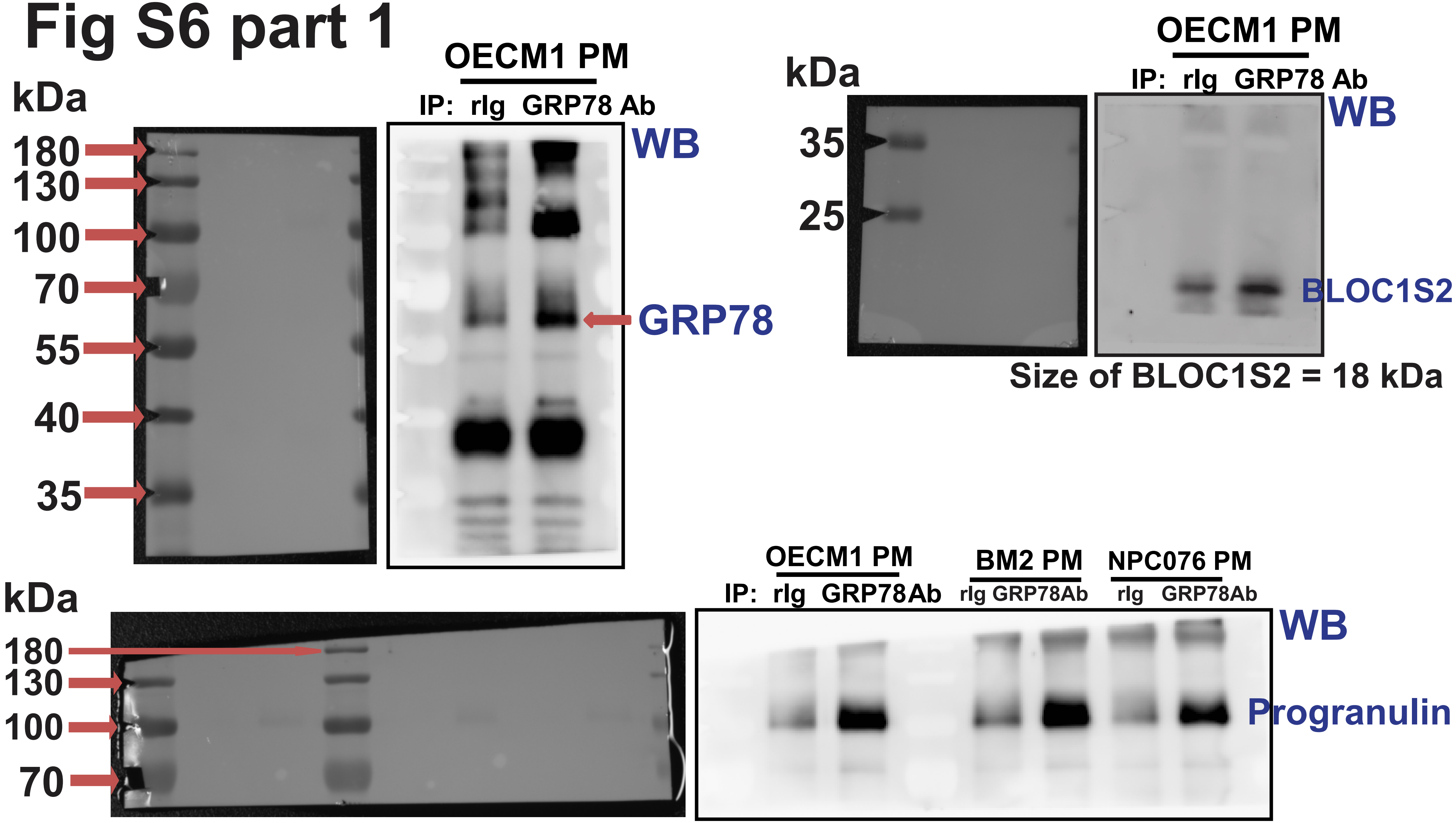

These three blots are the uncropped images of the blots in Fig 2B and 2D.

Fig S6 part 2

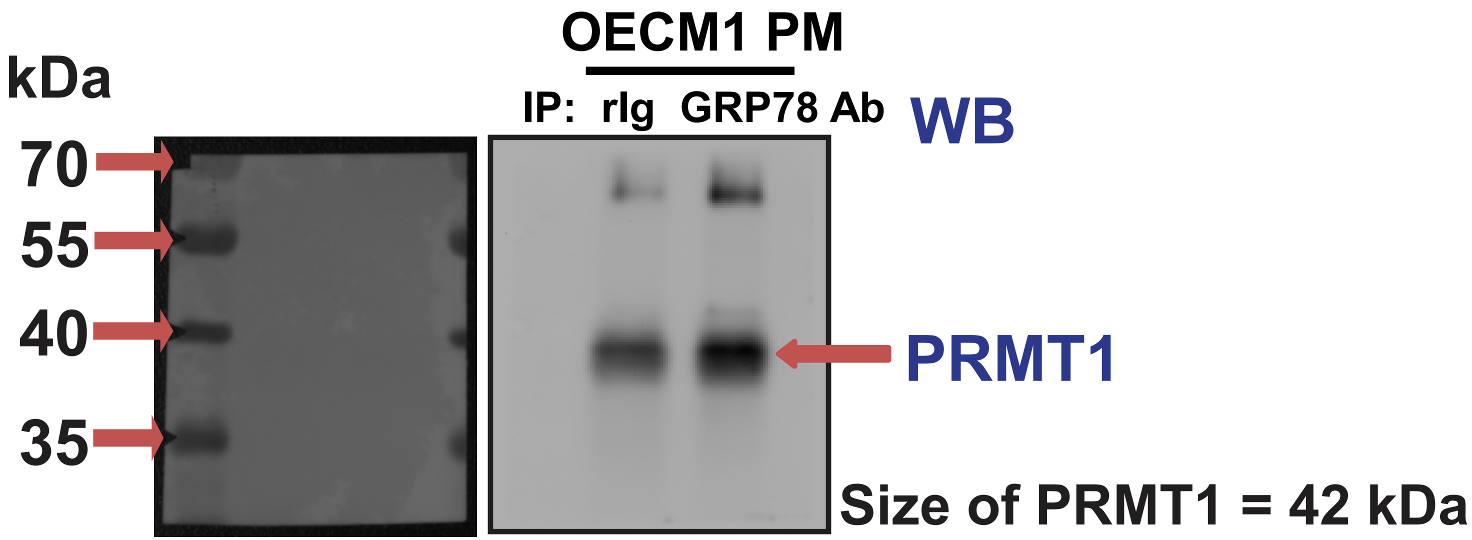

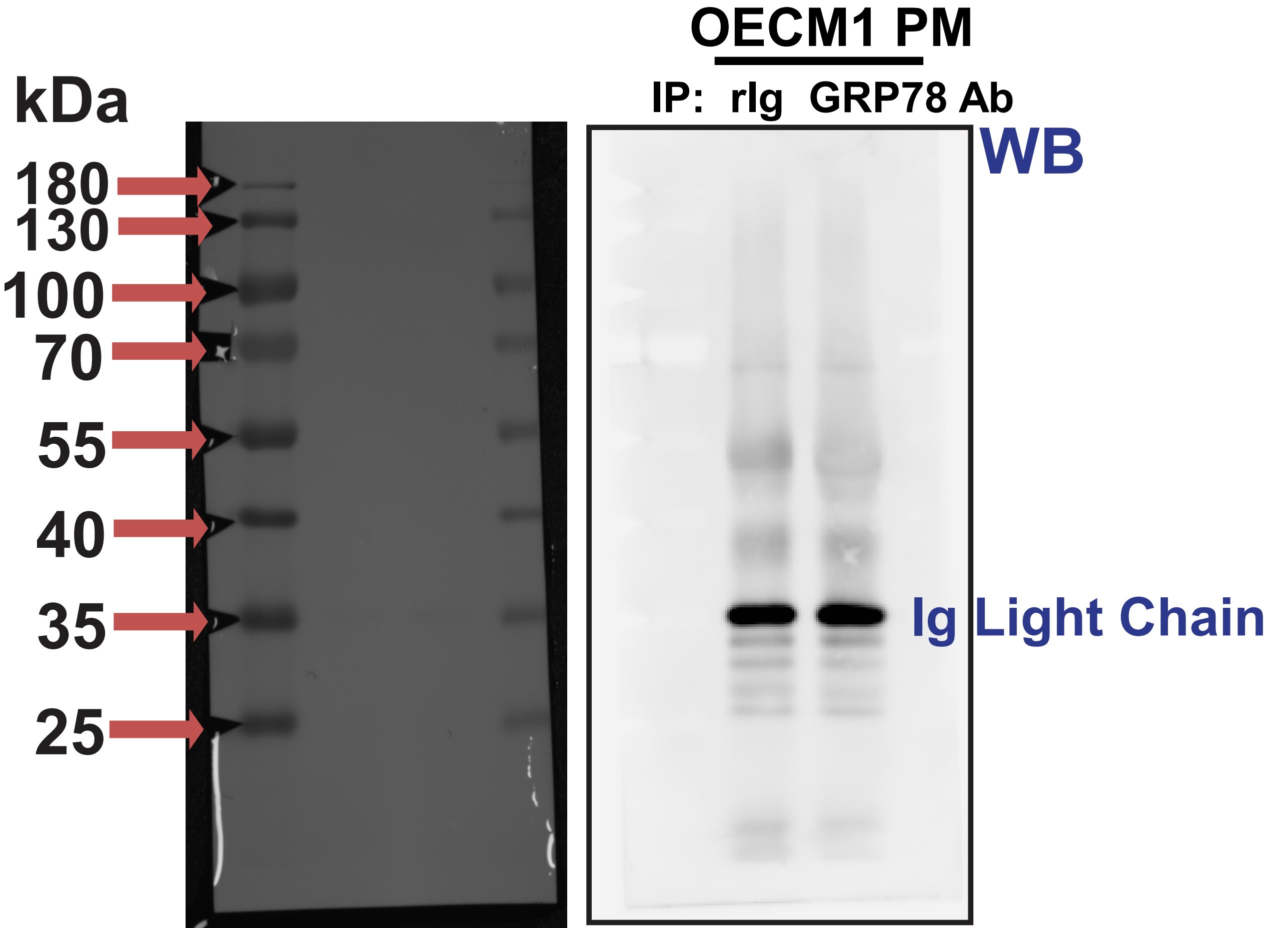

These two blots are the un-cropped images of the blots in Fig 2B.

Fig S7 part 1

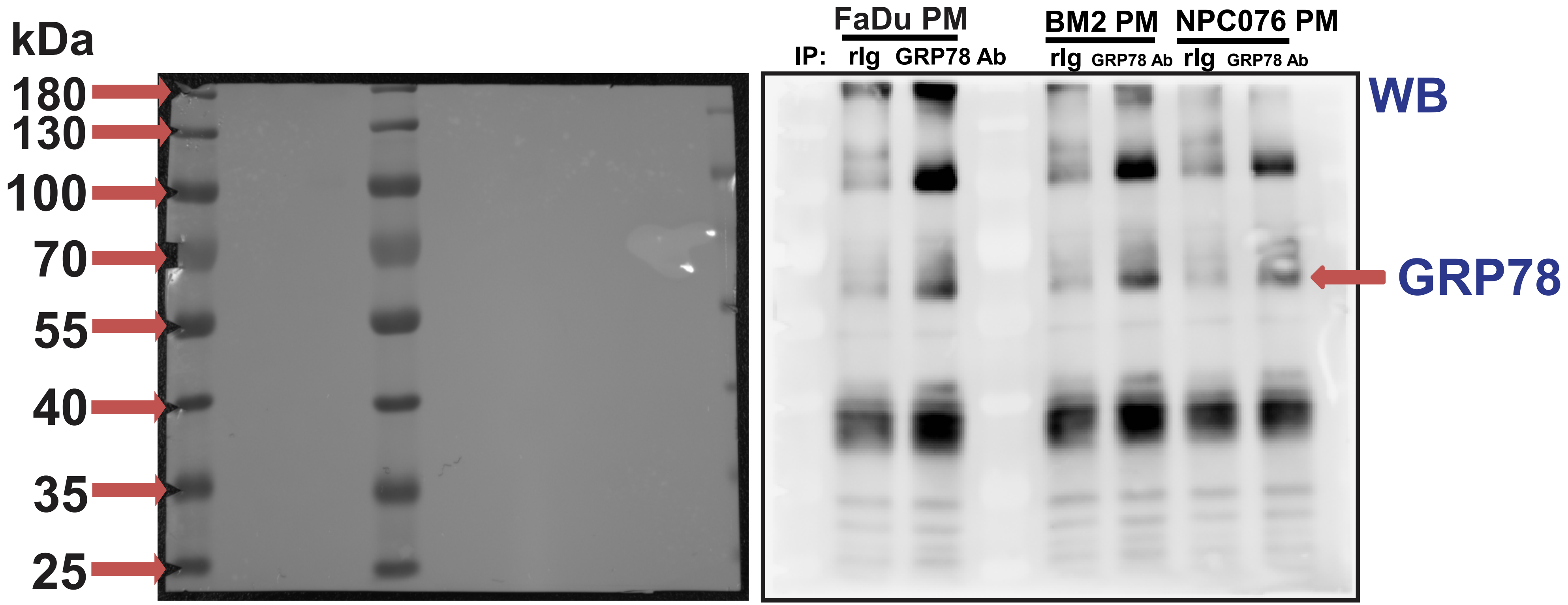

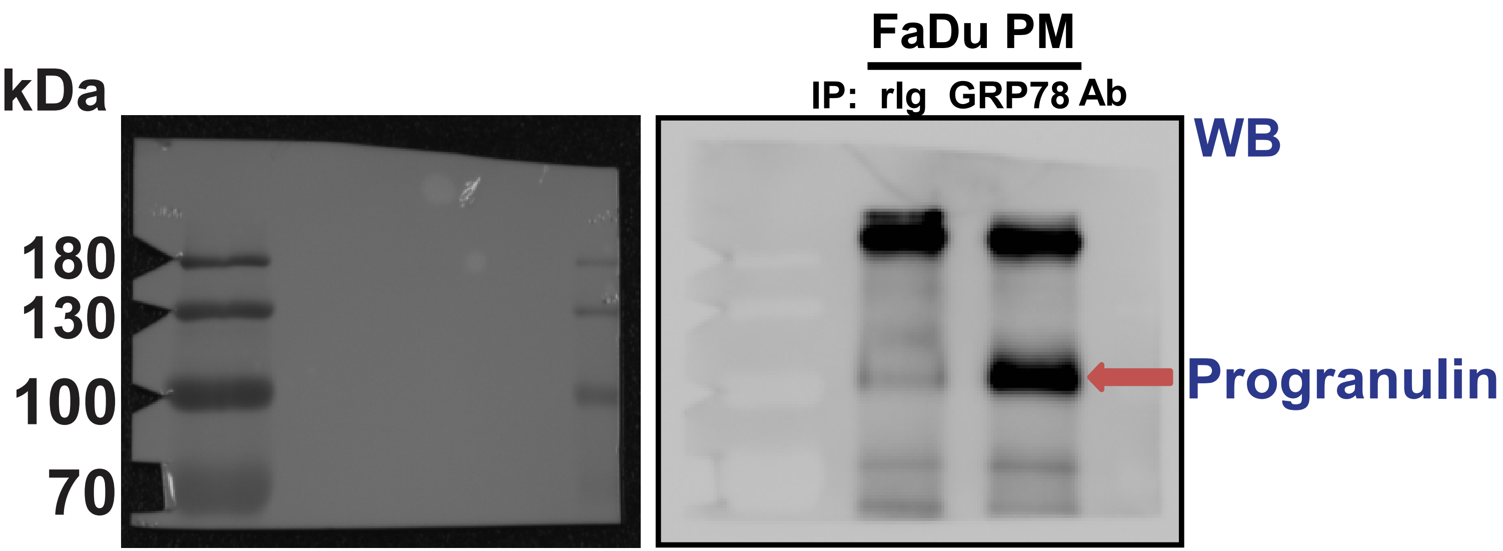

These two blots are the uncropped images of the blots in Fig 2C and 2D.

Fig S7 part 2

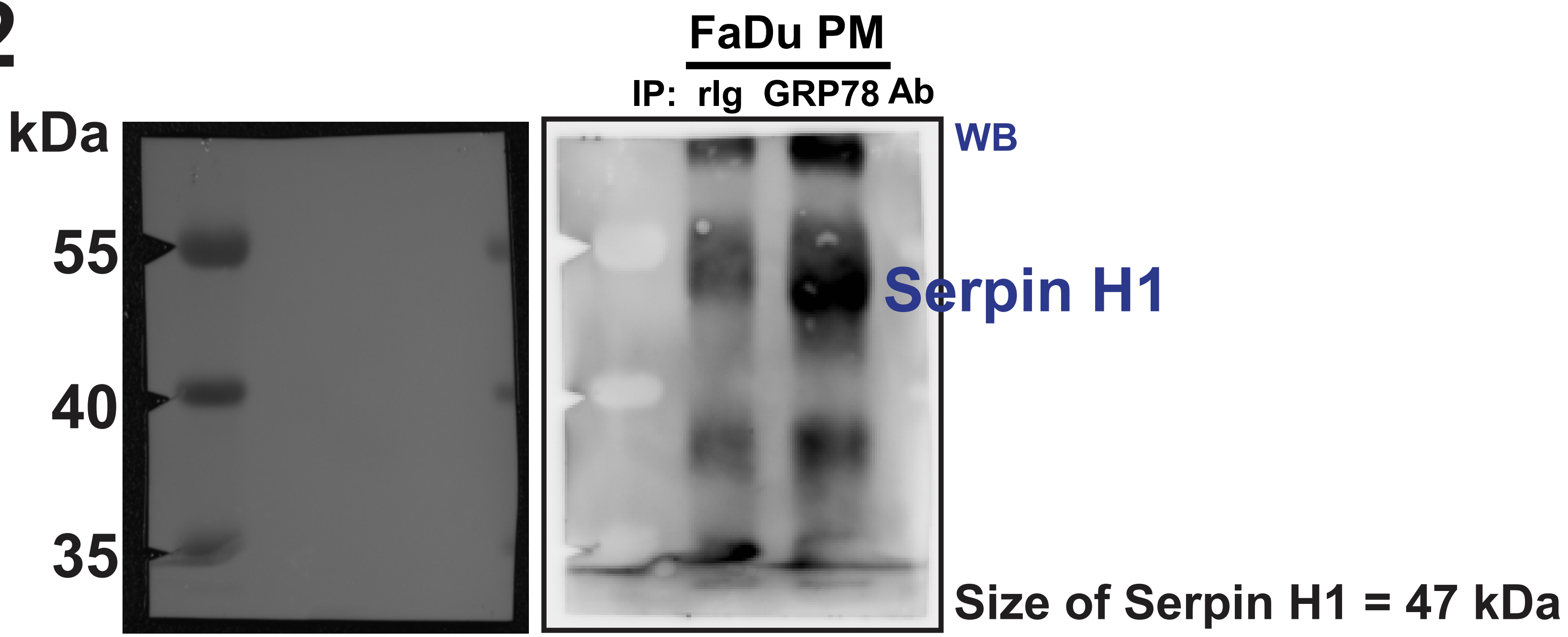

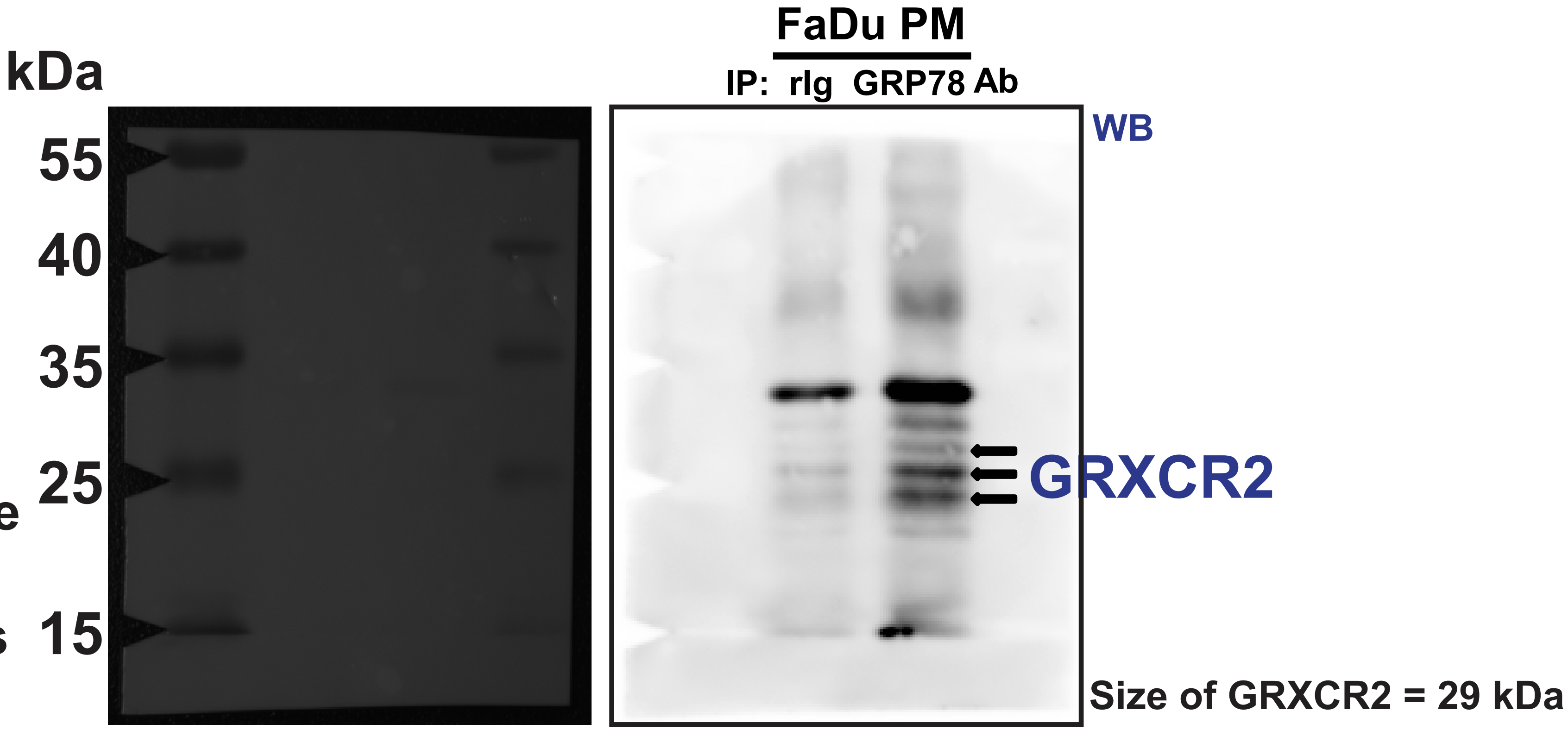

These two blots are the uncropped images of the blots in Fig 2C.

Fig S7 part 3

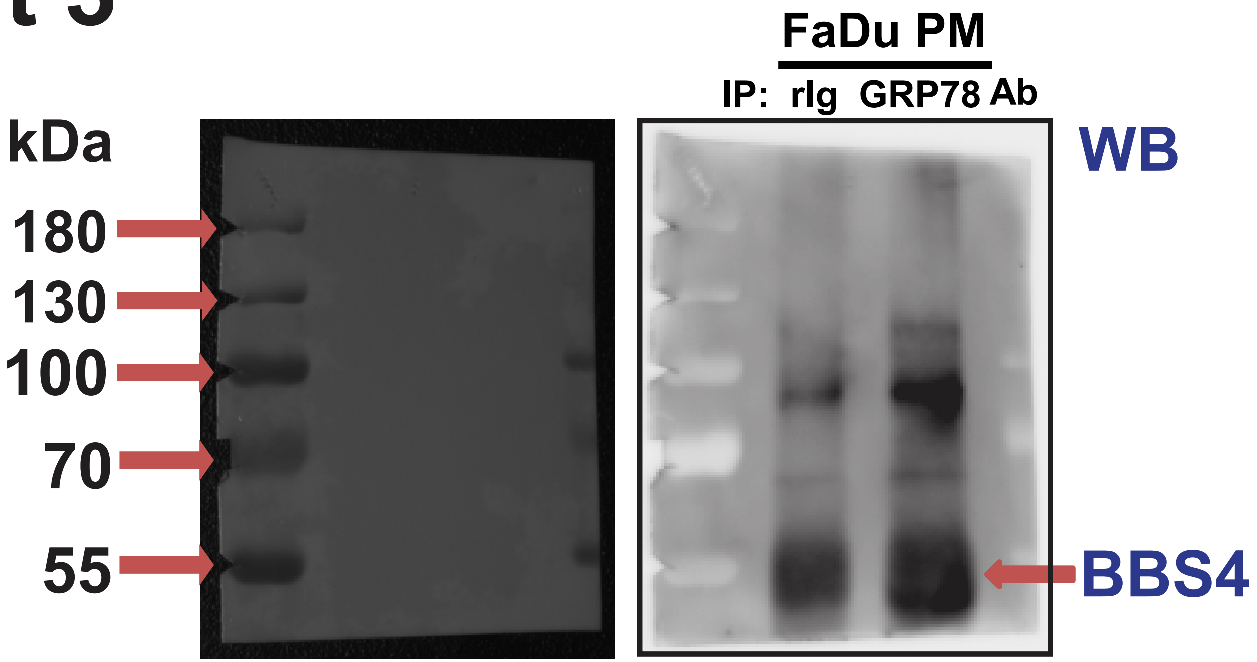

Size of BBS4 = 58 kDa

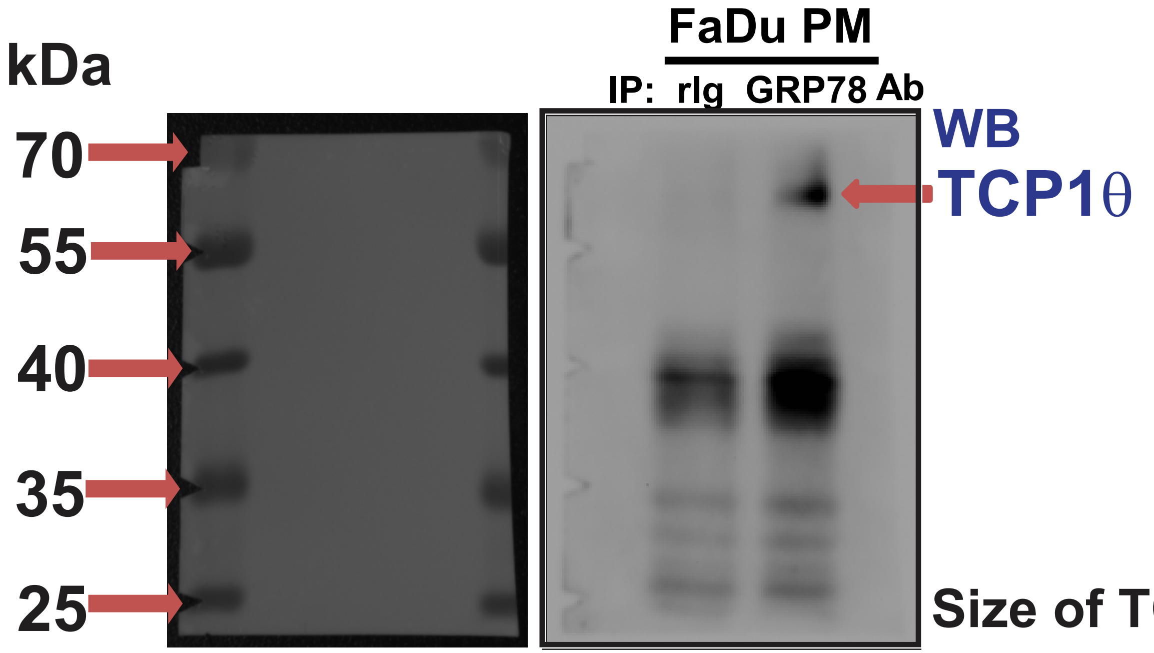

Size of TCP1θ = 52-65 kDa

These two blots are the uncropped images of the blots in Fig 2C.

Fig S7 part 4

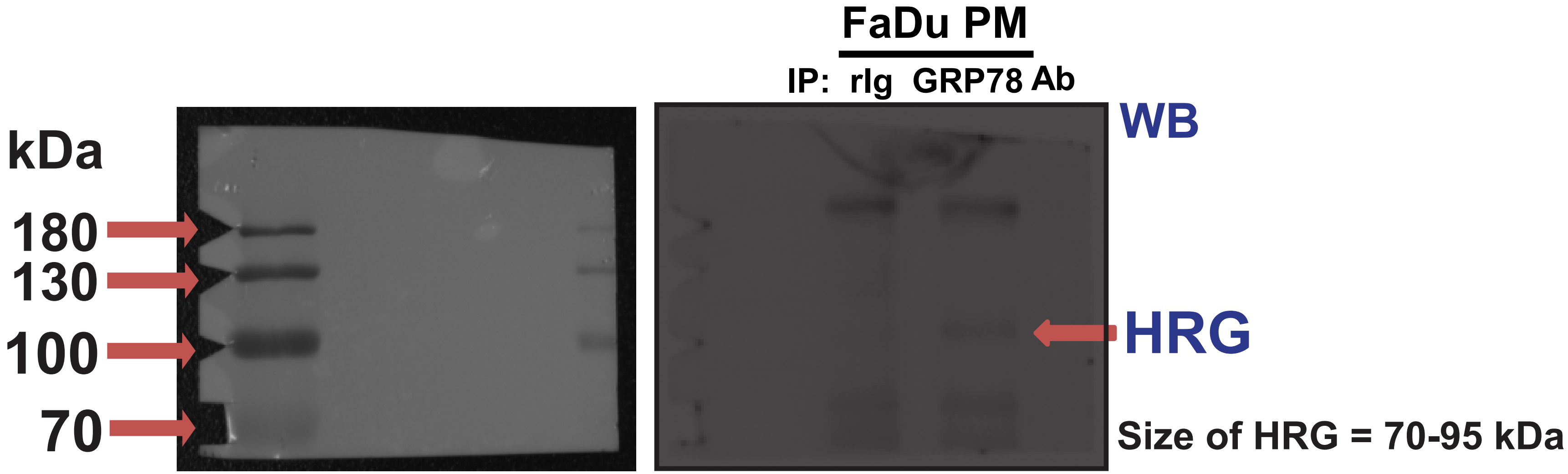

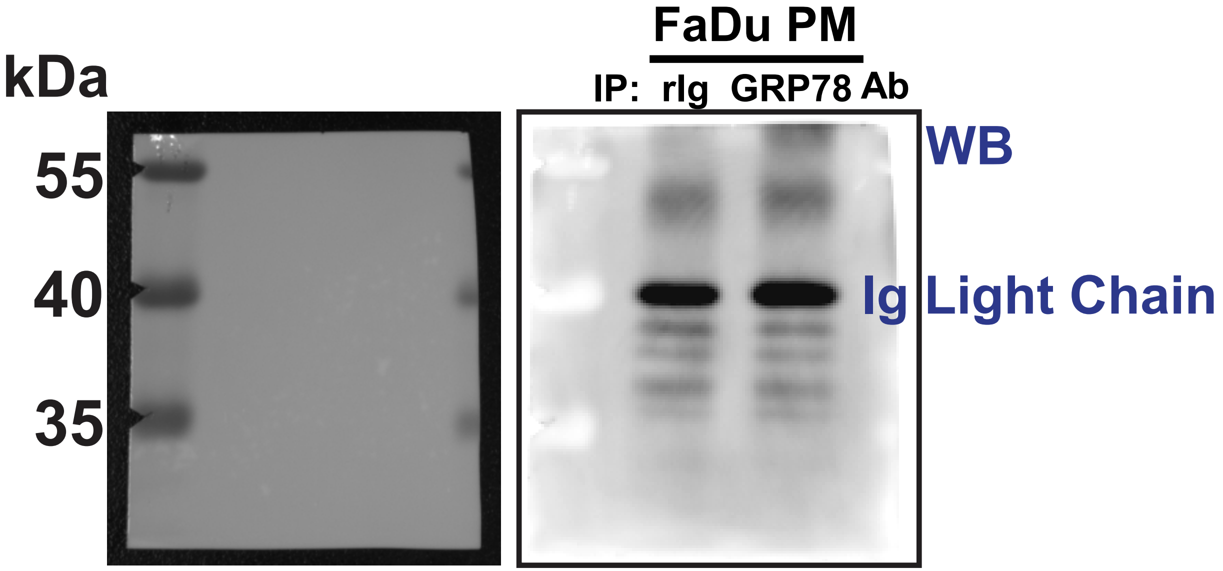

These two blots are the uncropped images of the blots in Fig 2C.

Fig S7 part 5

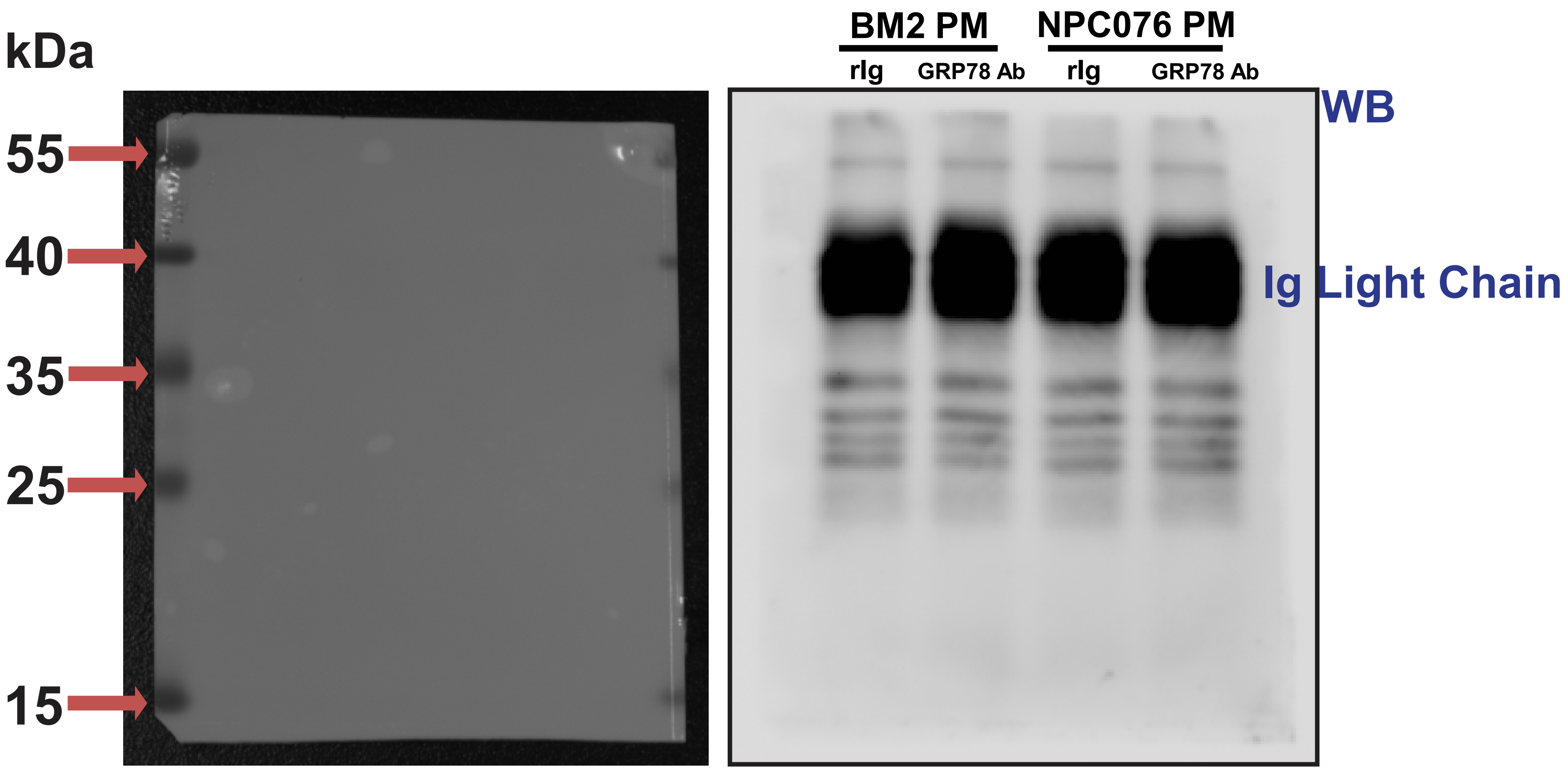

This blot is the uncropped image of one of the blots in Fig 2D.

Fig S8 part 1

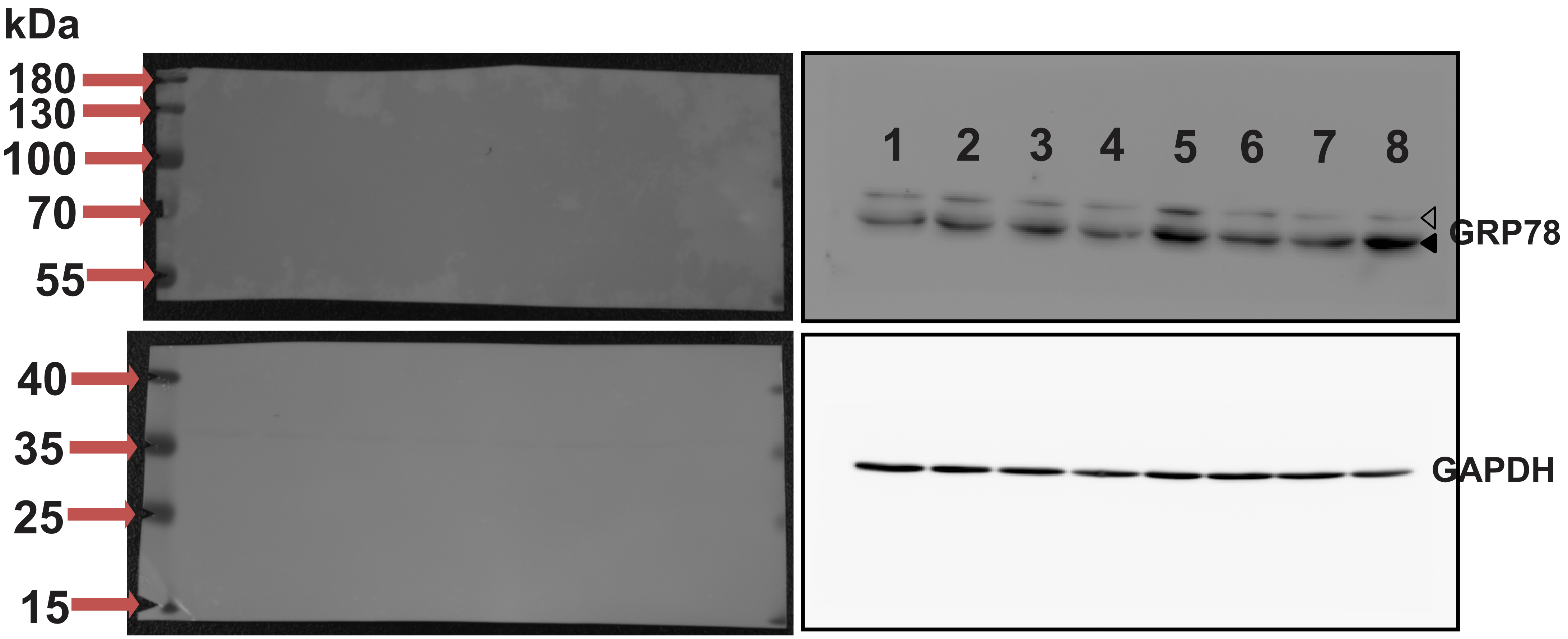

Lane 1: 5 μg Scramble plasmid, $3x10^5$ cells
Lane 2: 5 μg shGRP78 plasmid, $3x10^5$ cells
Lane 3: 6 μg Scramble plasmid, $3x10^5$ cells
Lane 4: 6 μg shGRP78 plasmid, $3x10^5$ cells
Lane 5: 5 μg Scramble plasmid, 10^6 cells
Lane 6: 5 μg shGRP78 plasmid, 10^6 cells
Lane 7: 6 μg Scramble plasmid, 10^6 cells
Lane 8: 6 μg shGRP78 plasmid, 10^6 cells

These two blots were cut from one membrane and are the the uncropped images of the blots in Fig S4. .
Open arrow head: cytosolic form of GRP78
Solid arrow head: membraneous form of GRP78

Fig S8 part 2

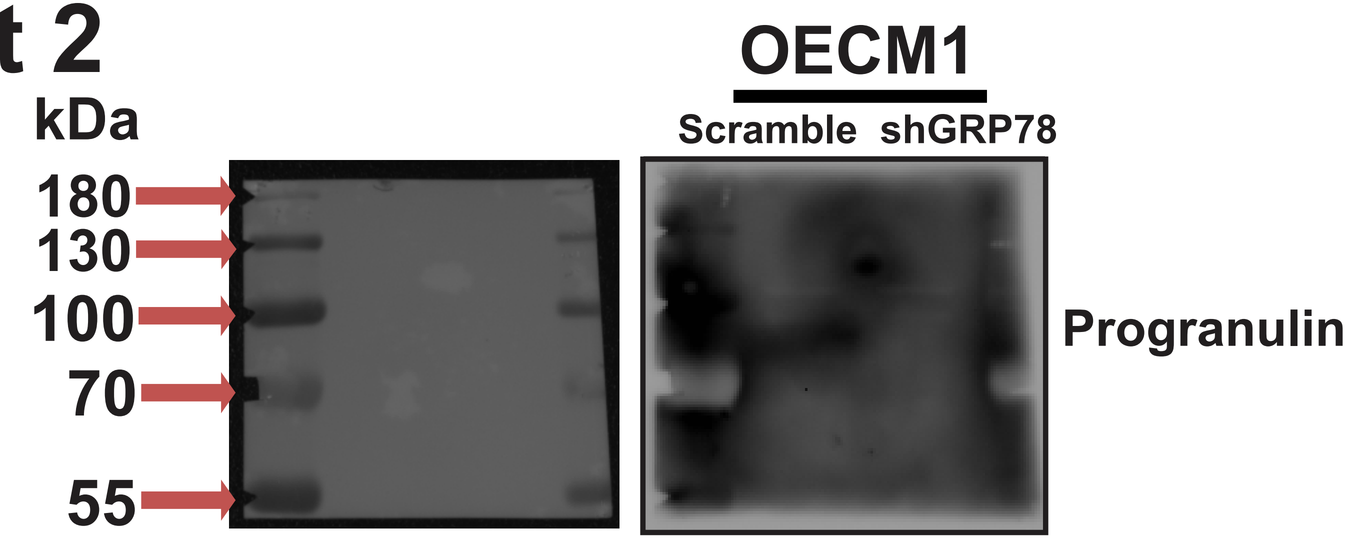

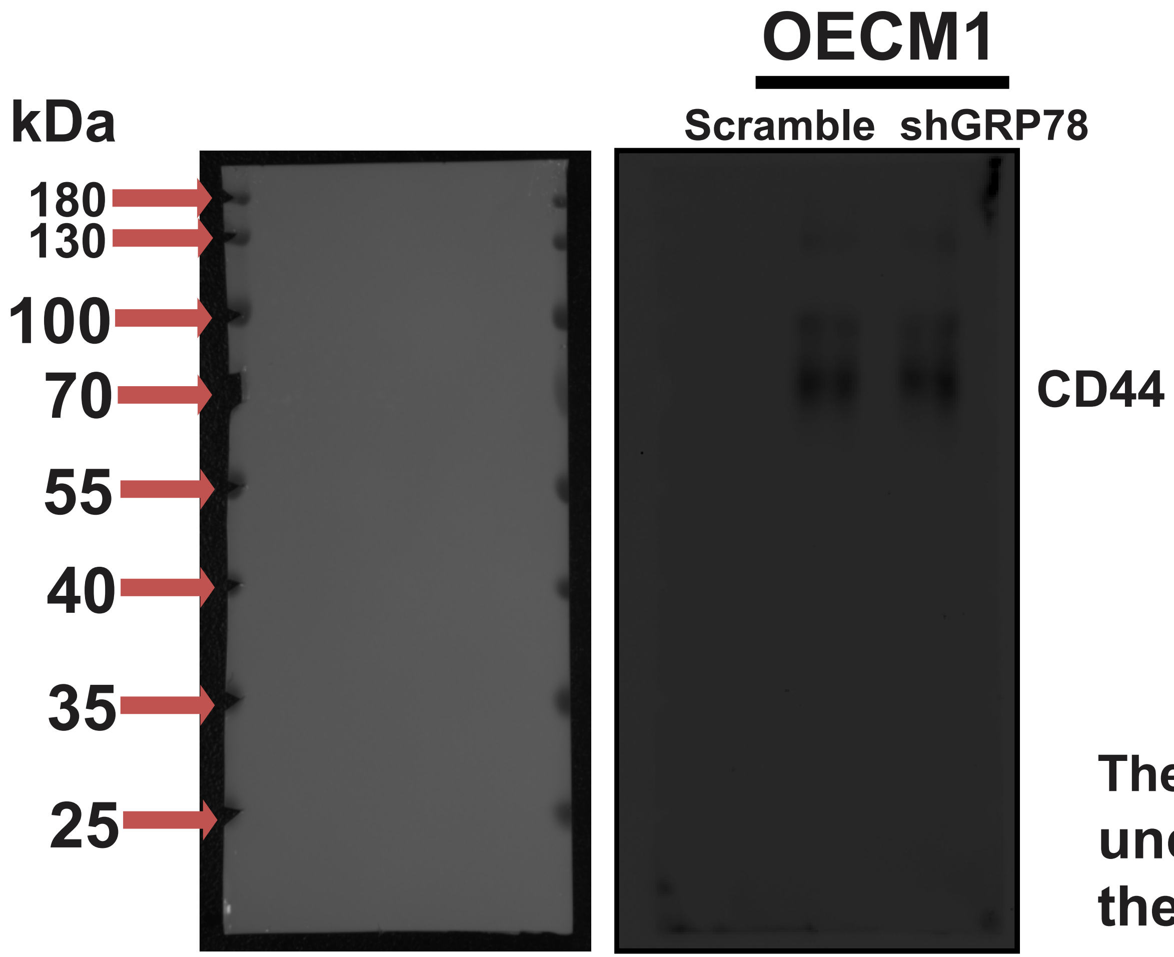

These two blots are the uncropped images of the blots in Fig S4.
